# Multi-omics analyses of evolved *Corynebacterium glutamicum* mutants reveal the molecular responses to formaldehyde stress

**DOI:** 10.1016/j.synbio.2026.01.020

**Published:** 2026-02-10

**Authors:** Liwen Fan, Qichen Cao, Zhihui Zhang, Xiaomeng Ni, Yu Lei, Tuo Shi, Jiuzhou Chen, Shengping Zhang, Wenjuan Zhou, Yu Wang, Ping Zheng, Jibin Sun

**Affiliations:** a Key Laboratory of Engineering Biology for Low-carbon Manufacturing, Tianjin Institute of Industrial Biotechnology, Chinese Academy of Sciences, Tianjin, 300308, China; b National Center of Technology Innovation for Synthetic Biology, Tianjin, 300308, China; c College of Biotechnology, Tianjin University of Science and Technology, Tianjin, 300457, China; d University of Chinese Academy of Sciences, Beijing, 100049, China

**Keywords:** Formaldehyde tolerance, *Corynebacterium glutamicum*, Adaptive laboratory evolution, Transcriptome analysis, Proteome analysis

## Abstract

Formaldehyde serves as a crucial intermediate metabolite during C1 compounds biotransformation. To optimize C1 utilization efficiency, it is essential to identify genes related to formaldehyde tolerance and enhance microbial resistance. Hence, we developed an evolved *Corynebacterium glutamicum* strain, FM-3, capable of withstanding 2.6 mM formaldehyde—a significant improvement over the parental strain. Integrated transcriptomic and proteomic analyses revealed that the enhanced formaldehyde tolerance correlated with the upregulation of cell wall biosynthesis proteins and DNA repair machinery. Genetic mutations identified in the evolved strains indicated that mutations in Cgl1199 (transcription termination factor Rho), and Cgl1590 (putative gluconeogenesis factor) played a pivotal role in formaldehyde tolerance. Further studies showed that Cgl1590 was involved in cell morphology regulation. This study enriches the understanding of formaldehyde tolerance mechanisms in *C. glutamicum* and provides guidance for enhancing strain tolerance to formaldehyde.

## 1 Introduction

Global climate change is one of the most pressing issues facing the world today. Recycling CO_2_ plays a vital role in reducing greenhouse gas emissions, which is crucial for achieving rapid and sustainable economic development. As a result, the biomanufacturing of chemicals and fuels from C1 feedstocks such as CO_2_ and methanol has attracted great attention recently [[1], [2], [3], [4]]. Representative C1 bioconversion processes include CO_2_-based chemoenzymatic synthesis of starch and polyhydroxyalkanoates [[4], [5], [6]] and microbial methanol fermentation using engineered natural or synthetic methylotrophs [[7], [8], [9], [10]]. In these processes, formaldehyde is generated as a key metabolic intermediate. As a ubiquitous metabolite in cellular metabolism, formaldehyde is also endogenously produced through demethylation reactions [11,12]. However, formaldehyde is a highly reactive and cytotoxic compound capable of interacting with biological nucleophiles in proteins and DNA, promoting the formation of DNA-protein cross-links [13]. The toxicity of formaldehyde seriously hinders the activity of mitochondrial respiratory enzymes, particularly NADH dehydrogenase (complex I) and cytochrome *c* oxidase (complex IV) [14], as well as cell metabolism and product synthesis [9]. For example, Chen et al. reported that the DNA-protein cross-links caused by formaldehyde resulted in an exceptionally prolonged lag phase of up to 20 days in methylotrophic *Escherichia coli* [15]. Similarly, Cai et al. found that formaldehyde accumulation severely impaired both cell growth and fatty acid production in engineered methylotrophic yeast strains [16]. Obviously, addressing the issue of formaldehyde toxicity and enhancing microbial tolerance to formaldehyde are essential for C1 bioconversion.

To mitigate the intracellular accumulation of excessive formaldehyde and its toxicity, many microorganisms are naturally equipped with a formaldehyde dissimilation pathway capable of rapidly oxidizing formaldehyde into CO_2_ [11,17,18]. However, the formaldehyde dissimilation pathway results in carbon loss as CO_2_, reducing the atomic economy of biosynthesis process and rendering carbon fixation efforts ineffective. Therefore, a common microbial metabolic engineering strategy in C1 bioconversion is to inactivate the dissimilation pathway and redirect formaldehyde into assimilation pathways [19,20]. Consequently, to enable efficient formaldehyde assimilation, developing a robust microbial chassis with enhanced formaldehyde tolerance becomes imperative to alleviate the associated cellular toxicity.

In addition to the formaldehyde dissimilation pathway, microorganisms have evolved different mechanisms to combat formaldehyde toxicity [[21], [22], [23]]. (*i*) The outer cell membrane served as the primary defense barrier against formaldehyde. Studies have shown that the formaldehyde tolerance of bacteria depended on the membrane composition and structure, and the presence of special outer membrane proteins was beneficial for improving the formaldehyde tolerance [21]. (*ii*) Beyond physical barriers, intracellular mechanisms also contribute to tolerance. The β-family nuclear transporter MSN5p in the cytoplasm was found to be associated with formaldehyde tolerance in *Candida boidinii*. It was speculated to mediate the nuclear import of replication protein A for DNA metabolism, including DNA replication, DNA repair, and recombination [22]. (*iii*) Formaldehyde can spontaneously react with cysteine to form thioproline, a proline analog that can be incorporated into peptides, resulting in aberrant peptides formation. The proline aminopeptidase (PepP) in *E. coli* can cleave these thioproline-containing peptides to improve the tolerance of strain to formaldehyde [23]. Although several genes have been identified as related to formaldehyde tolerance in microorganisms, in-depth study using multi-omics methods is still necessary to illustrate how formaldehyde affects global cellular metabolism and how cells respond to formaldehyde toxicity.

*Corynebacterium glutamicum*, a well-established industrial platform for amino acid and chemical production [24], has emerged as a promising chassis for synthetic methylotrophy in methanol bioconversion [10,[25], [26], [27]]. However, formaldehyde toxicity has been identified as a major bottleneck that limits the efficient methanol utilization and rapid cell growth on methanol [25,27].

In this study, we employed adaptive laboratory evolution (ALE) to enhance formaldehyde tolerance in *C. glutamicum* by subjecting a strain with an inactivated formaldehyde dissimilation pathway to progressively increasing formaldehyde stress. The resulting evolved mutants with significantly improved formaldehyde tolerance were screened. Comparative genomic, transcriptomic, and proteomic analyses were performed to uncover the genetic and regulatory adaptations underlying the improved formaldehyde resistance. Our findings not only advance the understanding of cellular responses to formaldehyde stress but also identify novel targets for rational metabolic engineering of formaldehyde-tolerant strains. This work contributes to overcoming the limitations imposed by formaldehyde toxicity in the bioconversion of C1 feedstocks, thereby facilitating their efficient industrial utilization.

## 2 Materials and methods

### 2.1 Bacterial strains and growth conditions

The strains used in this study are listed in Supplementary Table 1. *Escherichia coli* Trans1-T1 (TransGen Biotech, China) was used for general cloning. Cells were cultivated in Luria–Bertani (LB) broth at 37 °C with shaking at 220 rpm. Kanamycin (Km 50 μg/mL) or chloramphenicol (*Cm*, 20 μg/mL) was added according to the demand. Strain FM-1 (*C. glutamicum* ATCC 13032 Δ*adhE*Δ*ald*) and its derivatives were cultivated at 30 °C in TSB medium [28] or CGXII minimal medium [29] supplemented with 10 g/L glucose as the carbon source, and formaldehyde (0.8–2.6 mM) was added to provide a stress condition as required. The initial OD_600nm_ (optical density at 600 nm) was set as 0.1 for liquid culture. Meanwhile, the shake flasks were covered with a sealing membrane to avoid evaporation of formaldehyde. CGXII minimal medium plates containing 1.5 % agar were used to test formaldehyde tolerance of mutants. The overnight cultures were diluted to OD_600nm_ = 5 and gradually diluted (10^−1^ to 10^−7^) using CGXII minimal medium. Then, 2.5 μL of each dilution was spotted onto CGXII plates containing 10 g/L glucose as the carbon source with or without 1 mM formaldehyde. Colony growth was regularly observed during incubation at 30 °C.

### 2.2 ALE for enhancing formaldehyde tolerance

ALE of *C. glutamicum* strain FM-1 was performed using CGXII minimal medium supplemented with 10 g/L glucose as the carbon source and formaldehyde as the stress condition. Strain FM-1 was first cultivated in 250 mL shaking flasks with 50 mL TSB medium at 30 °C for 12 h, and then transferred to the CGXII medium with 0.8 mM formaldehyde at an initial OD_600nm_ of 0.1. When the OD_600nm_ of strain remained relatively constant, the culture was used as a seed to inoculate fresh medium with an initial OD_600nm_ of 0.1. Once the culture exhibited stable growth over consecutive passages at a given formaldehyde concentration, the formaldehyde concentration was gradually increased by 0.2 mM. Cells cultivated with resistance to 2.6 mM formaldehyde were diluted and spread on CGXII plates supplemented with 2.0 mM formaldehyde and 10 g/L glucose. Colonies with larger sizes were selected for subsequent test. Formaldehyde consumption was regularly detected by Nash reagent. Nash reagent was prepared with 2 M ammonium acetate, 50 mM acetic acid, and 20 mM acetylacetone (pH 7.2). Cells were cultivated in 250 ml shaking flasks with 50 mL CGXII medium supplemented with 10 g/L glucose and 0.8 mM formaldehyde at 30 °C. The OD_600nm_ and formaldehyde consumption were measured at regular intervals. Nash assay was started by mixing 20 μL cell culture supernatant and 180 μL Nash reagent. The mixture was incubated at 65 °C for 20 min, and then OD_412nm_ measurement was taken.

### 2.3 Transcriptome analysis

The starting strain FM-1 and the evolved mutant FM-3 that exhibited significantly enhanced formaldehyde tolerance were cultivated in CGXII minimal medium supplemented with or without 0.8 mM formaldehyde at 30 °C and with shaking at 220 rpm, and 10 g/L glucose was supplemented as the carbon source. To capture dynamic transcriptional responses when gene regulation is most active and mRNA levels fluctuate rapidly, cells were harvested at the mid-exponential phase for RNA isolation. The sample size (N, number of biological replicates) of FM-1 and FM-3 was 3. RNA preparation, library construction, transcriptome sequencing and analysis were performed according to the procedure described previously [25]. Cell disruption was achieved using 0.1 mm zirconia beads in a high-throughput cryogenic grinder, followed by total RNA extraction with the Magnetic Tissue/Cell/Blood Total RNA Kit (Tiangen). Quantification of the RNA concentration was carried out using a Qubit 2.0 fluorometer, while the integrity of the RNA was evaluated through agarose gel electrophoresis.

For transcriptome library construction, ribosomal RNA was first removed from 500 ng of total RNA using the QIAseq FastSelect-5S/16S/23S Kit (Qiagen) according to the manufacturer's protocol. RNA-seq libraries were then prepared using the NEBNext® Ultra II Directional RNA Library Prep Kit for Illumina (NEB). The concentration of the library was determined using a Qubit 2.0 fluorometer, and its fragment distribution profile was analyzed with a 2100 Bioanalyzer. The qualified libraries were sequenced by Novogene (Beijing, China). To ensure high-quality data for downstream transcriptomic analysis, the raw reads were subjected to quality control and filtering using FastP (version 0.23.1), which included the removal of adapter sequences and low-quality bases. The filtered data were further processed with the DESeq function of the DeSeq2 package (version 1.18.1) to analyze differentially expressed genes. After that, genes with a false discovery rate (FDR) ≤0.05 and log_2_(Fold change) ≥1 or ≤ −1 were considered to be differentially expressed. Subsequent functional annotation was performed using the KEGG Automatic Annotation Server (KAAS) (https://www.genome.jp/kegg/kaas/) [30]. Pearson's linear correlation coefficients between variables were calculated using the R package ‘stats’ and plotted using ‘corrplot’. Principal component analysis was performed using ‘stats’ package and plotted using ‘ggord’ package. All R scripts and input data used in this study are available at https://github.com/YuWangLab/formaldehyde-tolerance.

### 2.4 Proteome analysis

The starting strain FM-1 and the evolved mutant FM-3 were cultivated in CGXII minimal medium supplemented with or without 0.8 mM formaldehyde at 30 °C and with shaking at 220 rpm, and 10 g/L glucose was supplemented as carbon source. Considering the temporal lag between transcript and protein abundance, cells were harvested at the late exponential phase and washed twice with 50 mM sodium phosphate buffer (pH 7.4). The sample size (N**,** number of biological replicates) of FM-1 and FM-3 was 3. For total protein extraction, the lysis of cells was performed by beads beating with FastPrep®-24 Classic Instrument (MP Biomedicals, CA, USA). The lysis buffer contained of 8 M urea, 50 mM Tris-HCl and 1 × protease cocktail (pH 8). The cell lysate was centrifuged at 15,000×*g* for 5 min at 4 °C and the supernatant was collected. The protein concentration was determined by Pierce™ Rapid Gold BCA Protein Assay Kit (Thermo Fisher Scientific, USA). One aliquot of the protein samples was subjected to SDS-PAGE analysis, the protein band was excised from the SDS-PAGE gel and cut into small piece. In-gel digestion was performed as described previously [31]. Briefly, the gel pieces were destained with 100 mM ammonium bicarbonate/acetonitrile (1:1, v/v), followed by reduction with dithiothreitol (DTT) and alkylation with iodoacetamide (IAM). The gel pieces were dehydrated with acetonitrile, rehydrated in trypsin solution, and incubated overnight at 37 °C for digestion. The resulting peptides were extracted with acetonitrile, dried under vacuum, dissolved in 0.1 % formic acid, and subjected to LC–MS/MS analysis. The other samples were subjected to thiol reduction, alkylation, and tryptic digestion following the filter-aided sample preparation (FASP) procedure with minor modifications [32]. Before MS analysis, iRT peptides [33] were spiked into the *C. glutamicum* peptide sample. The nanoLC-MS/MS analysis was performed on an Eksigent NanoLC connected to the TripleTOF 5600 mass spectrometer (AB SCIEX, Concord, Ontario) with a nano-electrospray ionization source. The SWATH-MS technique [34] was implemented for high-throughput quantitative analysis. Wiff files from mass spectrometry were converted to mzXML format by ProteoWizard MSconvert [35] (version 2.6.0). The targeted XICs extraction was performed by the OpenSWATH [36,37] (version 2.7.0) software against the previously generated spectra library. Statistical validation and multirun alignment were conducted by the tool PyProphet [38] and TRIC software [39] with default settings [37]. The proteome absolute quantitation was conducted by previously developed nMAQ approach [40], proteins of the target (^14^N) sample and the (^15^N) reference sample were 1:1 mixed and followed by SWATH-MS analysis. The resulting intensities were used to calculate the iBAQ values of the ^14^N and ^15^N labeled proteins. The regression curve was generated with the iBAQ values versus the pre-quantified amount of the heavy “anchor proteins”. The iBAQ values of the target (^14^N) proteins were used to calculate the absolute contents from the regression curve. The differential expression analysis was conducted using Perseus (*ver*. 2.0.3.1) software [41] and the proteins with false discovery rate (FDR) ≤0.05 and log_2_(Fold change) ≥1 or ≤ −1 were considered to be differentially expressed. Functional annotation was performed using KAAS (https://www.genome.jp/kegg/kaas/) [30]. Pearson's linear correlation coefficients and PCA were conducted using the same methods as those applied to the transcriptomic data. All R scripts and input data used in this study are available at https://github.com/YuWangLab/formaldehyde-tolerance.

### 2.5 Whole genome sequencing

Whole genome sequencing and analysis of mutations introduced by ALE were performed as previously described [27]. Genomic DNAs of evolved *C. glutamicum* strains were extracted by Wizard Genomic DNA Purification Kit (Promega (Beijing) Biotech Co., China). BioMarker (Beijing, China) company carried out the corresponding library construction and sequencing work, and Illumina Hiseq2500 sequencing platform was adopted in the work. Quality assurance of the output was performed using FastQC software (version 0.10.1) and NGSQC Toolkit software (version 2.3.3). Comparative analysis and variant calling were based on BWA alignment software (version 0.7.17) and SAM tools software (version 1.9). The annotations for variations were made by using the SnpEff software (version 4.3i).

### 2.6 Genetic modification of *C. glutamicum*

The suicide plasmid pK18*mobsacB* [42] was used for integrating the nucleotide mutation into FM-1. The plasmids used in this study and primers for plasmid construction were listed in Supplementary Table 1 and Supplementary Table 2, respectively. Taking the integration of the *cgl1590*^750insG^ mutation into the FM-1 chromosome as an example, a plasmid pK18-*cgl1590*^750insG^ containing a ∼2 kb mutant fragment of *cgl1590* was constructed. The mutant *cgl1590* fragment was amplified from the genomic DNA of *C. glutamicum* ATCC 13032 using the primer pairs *cgl1590*^750insG^-F1/*cgl1590*^750insG^_-_R1, *cgl1590*^750insG^-F2/*cgl1590*^750insG^-R2, and then ligated with the *Bam*HI-linearized pK18*mobsacB* using the ClonExpress MultiS One Step Cloning Kit (Vazyme Biotech, China). Primer synthesis and Sanger sequencing were performed by AZENTA (China). The resultant plasmid pK18-*cgl1590*^750insG^ was transferred into FM-1 via electroporation for allelic exchange, generating the mutant strain FM-1-*cgl1590*^750insG^. A similar procedure was performed to integrate the rest nucleotide mutations, generating the corresponding mutants.

Another three nucleotide mutations (*cgl0752*^G761T^, *cgl0942*^G443T^, and *cgl1199*^1015−1032del^) were constructed by using the CRISPR/Cas9-mediated ssDNA recombineering [43]. Taking introduction of the *cgl0752*^G761T^ mutation as an example, pCas9gRNA-*cgl0752* was constructed by Golden Gate assembly of pCas9gRNAccdB plasmid [28] and the annealed double-stranded DNA of the primer pair *cgl0*752-gF/gR. The pCas9gRNA-*cgl0752* and the ssDNA containing *cgl0752* ^G761T^ mutation were co-transformed into *C. glutamicum* ATCC 13032 (pRecT) via electroporation. *cgl0752* edited mutants were screened following the procedure described previously [28]. *cgl0942*^G443T^ and *cgl1199*^1015−1032del^ nucleotide mutations were introduced following a similar procedure. The knockout of the target gene was carried out following the aforementioned gene knock-in procedure using pK18*mobsacB*. Target genes were overexpressed in *C. glutamicum* using the *E. coli*-*C. glutamicum* shuttle vector pEC-XK99E [44]. pEC-XK99E-*cgl1590* was obtained from a genome-scale open reading frame library of *C. glutamicum* ATCC 13032, which was constructed by Tianjin Institute of Industrial Biotechnology [7]. The plasmids were transformed into FM-1 by electroporation. Expression of the target genes was controlled by an isopropyl β-d-thiogalactoside (IPTG)-inducible promoter P_*trc*_, and 0.1 mM IPTG was added to the culture for inducing gene overexpression.

### 2.7 Scanning Electron Microscope (SEM) analysis

Strains were grown in CGXII minimal medium supplemented with 10 g/L glucose, in the presence or absence of 0.8 mM formaldehyde. Cells in the middle exponential phase were collected and washed with potassium phosphate buffer (pH 7.4). Subsequently, cells were immersed in 2.5 % glutaraldehyde fixative and incubated overnight at 4 °C. After fixation, Cells were resuspended by 1 % osmic acid for 1h at 4 °C. The intracellular water was gradually replaced with a graded ethanol series for dehydration. After that, the samples were subjected to critical point drying using liquid carbon dioxide. Then, the dried samples were mounted on adhesive tape and coated with platinum (Pt). Finally, the samples were observed and imaged by a scanning electron microscope (Hitachi SU8010, Japan). Cell length distribution was determined by measuring 70 cells of each strain and analyzed using Image J software.

### 2.8 Structure prediction of Cgl1199 and its mutant using AlphaFold3

The structures of the Cgl1199 and its mutant protein were predicted using AlphaFold3 (https://alphafoldserver.com/) [45]. The full-length amino acid sequence was submitted to the AlphaFold3 server. The prediction was carried out under default parameters, and the resulting 3D structure was visualized and analyzed using PyMOL to assess potential structural changes induced by the deletion of six amino acid residues (RNRRGR) corresponding to positions 339 to 344.

## 3 Results

### 3.1 Evolving *C. glutamicum* for improved formaldehyde tolerance

**Fig. 1:**
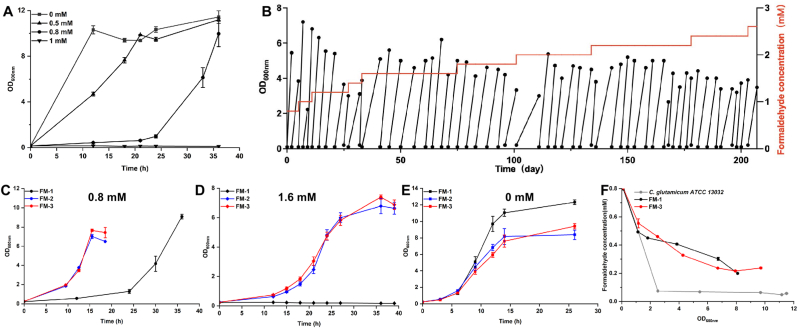
Improving the tolerance to formaldehyde via ALE. (A) Growth of FM-1 in minimal medium with 10 g/L glucose and different formaldehyde concentrations. 0 mM (square), 0.5 mM (triangle), 0.8 mM (circle), and 1 mM (inverted triangle). (B) ALE procedure of culture-1 in CGXII minimal medium supplemented with different formaldehyde concentrations and 10 g/L glucose. (C) Growth curve of the evolved mutants in CGXII minimal medium supplemented with 10 g/L glucose and 0.8 mM formaldehyde. (D) Growth curve of evolved mutant in CGXII minimal medium supplemented with 10 g/L glucose and 1.6 mM formaldehyde. (E) Growth curve of evolved mutant in CGXII minimal medium supplemented with 10 g/L glucose. (F) Formaldehyde degradation during cell growth of wild-type *C. glutamicum* ATCC 13032, FM-1 and FM-3. Values and error bars reflect the mean ± s.d. of three biological replicates (N = 3).

*C. glutamicum* ATCC 13032 possesses an endogenous formaldehyde dissimilation pathway. Previous studies have demonstrated that the oxidation of formaldehyde to formate relies on two enzymes: acetaldehyde dehydrogenase (Ald) and mycothiol-dependent formaldehyde dehydrogenase (AdhE) [18,46]. Ald directly catalyzes the oxidation of formaldehyde to formate. In contrast, AdhE functions through a two-step mechanism. First, formaldehyde spontaneously conjugates with mycothiol (MSH) to produce *S*-(hydroxymethyl) mycothiol. This intermediate is then oxidized by AdhE to *S*-formylmycothiol, which is subsequently hydrolyzed to formate and MSH. To assess the toxicity of formaldehyde in *C. glutamicum* ATCC 13032 lacking the formaldehyde dissimilation pathway, we cultivated a previously developed *C. glutamicum* FM-1 strain (*C. glutamicum* ATCC 13032 Δ*adhE*Δ*ald*), which was referred to as MX-1 in our previous study [27], in CGXII medium supplemented with various concentrations of formaldehyde (Fig. 1A). All shake flasks were equipped with sealing membranes to prevent the evaporation of formaldehyde (Supplementary Fig. 1). The growth of FM-1 was affected primarily in the lag phase with increasing formaldehyde concentration. When 0.5 mM or a higher concentration of formaldehyde was added, an extended lag phase was observed. The FM-1 strain showed no detectable growth at 1.0 mM formaldehyde after 36 h of cultivation. These results are consistent with previous reports that growth of the wild-type *C. glutamicum* with inherent formaldehyde detoxification pathway is severely inhibited at 1 mM formaldehyde [18].

ALE is an effective strategy for improving the phenotypes associated with cell growth, including the cellular tolerance to environmental stress [47]. In this study, strain FM-1 with deactivated formaldehyde dissimilation pathway was selected as the starting strain for ALE to enhance the tolerance to higher concentrations of formaldehyde. Based on preliminary tolerance assays, 0.8 mM formaldehyde was selected as the initial stressor, with incremental increases of 0.2 mM upon culture adaptation. Two parallel ALE experiments were conducted. One experiment continued for 207 days and 56 passages (≈296 generations) (designated as ALE culture-1), reaching a final formaldehyde concentration of 2.6 mM (which was over threefold higher than the initial level. At this concentration, cultures achieved final OD_600_ values of approximately 2.0–3.0 (Fig. 1B). The parallel experiment continued for 203 days and 57 passages (≈299 generations) (designated as ALE culture-2). However, the final OD_600nm_ values of the ALE cultures were approximately 1.0 under 2.6 mM formaldehyde (Supplementary Fig. 2A), likely due to the high toxicity of formaldehyde and the heterogeneous mutational trajectories acquired by parallel ALE lineages.

The final ALE cultures were diluted and plated on CGXII plates containing formaldehyde. Two largest colonies (designated as FM-2 and FM-3) were isolated from the ALE culture-1 plates. Their growth performance was evaluated under different concentrations of formaldehyde, with the starting strain FM-1 as a control. In the presence of 0.8 mM formaldehyde, the evolved strains FM-2 and FM-3 exhibited faster growth during the exponential phase and a markedly shorter lag phase compared to FM-1. The specific growth rate during exponential phase of FM-2 and FM-3 reached 0.22 h^−1^ and 0.23 h^−1^, which were 1.37- and 1.44-fold higher than that of FM-1 (0.16 h^−1^), respectively (Fig. 1C).When exposed to a higher concentration of formaldehyde (1.6 mM), the evolved strains FM-2 and FM-3 resumed growth after an approximate 10-h lag phase, whereas the parental strain FM-1 exhibited no detectable growth (Fig. 1D). This confirms the successful acquisition of enhanced formaldehyde resistance through adaptive evolution. Interestingly, under non-stress conditions, the evolved strains exhibited slower exponential growth than FM-1 (Fig. 1E), suggesting that mutations conferring formaldehyde resistance impose a fitness cost in the absence of stress. Another six evolved mutants (designated as FM-4 to FM-9) were isolated from the parallel ALE culture-2. Although five of them could grow under 1.6 mM formaldehyde, unlike the non-growing FM-1, none outperformed FM-2 and FM-3 in terms of formaldehyde tolerance (Supplementary Fig. 2B and Fig. 1D).

Although the formaldehyde dissimilation pathway was blocked in FM-1, the enhanced tolerance of the evolved mutants could potentially arise from other evolved formaldehyde degradation mechanisms. To exclude such possibility, the formaldehyde degradation of the evolved strain FM-3, the starting strain FM-1, and the wild-type *C. glutamicum* were determined. Cells were cultivated in CGXII minimal medium containing 0.8 mM formaldehyde and 10 g/L glucose, and formaldehyde concentrations were monitored using the Nash reagent (Fig. 1F). These results confirm that the improved tolerance of FM-3 (Fig. 1D) was independent of formaldehyde degradation. The underlying mechanisms of formaldehyde tolerance in FM-3 were therefore investigated in subsequent experiments. The wild-type *C. glutamicum* showed rapid formaldehyde degradation rate due to its native formaldehyde dissimilation pathway, whereas FM-3 displayed a markedly lower degradation rate, comparable to that of FM-1. These results conclusively demonstrate that the acquired tolerance in FM-3 (Fig. 1D) stems from cellular adaptation rather than formaldehyde detoxification, prompting subsequent investigation of its resistance mechanism.

### 3.2 Identifying the key mutations endowing cellular tolerance to formaldehyde

**Fig. 2:**
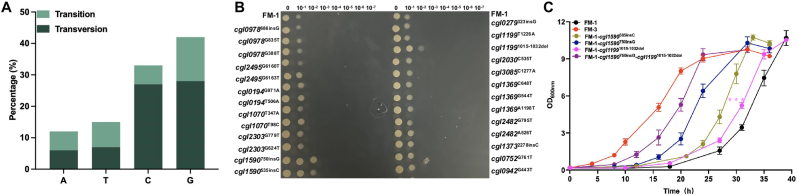
Effects of single-site mutations on formaldehyde tolerance. (A) Frequency of mutations of four bases in evolved strain FM-3. (B) Growth of strain FM-1 and its derivatives harboring single-site mutations on CGXII minimal agar medium supplemented with 10 g/L glucose and 1 mM formaldehyde. (C) Growth curves of strain FM-1 and its derivatives in CGXII medium supplemented with 10 g/L glucose and 0.8 mM formaldehyde. Values and error bars reflect the mean ± s.d. of three biological replicates (N = 3). Statistical significance at 31 h between FM-1-*cgl1199*^1015−1032del^ and FM-1 was determined by unpaired two-tailed Student's *t*-test: ∗∗∗P < 0.001.

To identify the mutations accumulated during ALE that conferred enhanced formaldehyde tolerance, the genomes of four evolved mutants FM-2, FM-3, FM-4, and FM-5 were sequenced. Each evolved strain possesses more than 100 mutations including single nucleotide polymorphisms (SNPs), insertions, and deletions, which were aligned against the *C. glutamicum* ATCC 13032 reference genome (GenBank accession number GCA_000011325.1). The high number of accumulated mutations may be attributed to severe DNA damage induced by formaldehyde toxicity, as error-prone repair mechanisms can lead to high-frequency mutagenesis [48]. Among the SNPs, base transversions of guanine (G) and cytosine (C) accounted for 51–66 % (FM-3 was shown in Fig. 2A, and FM-2, FM-4, FM-5 in Supplementary Fig. 3A, 3B and 3C, respectively). Frameshift mutations predominantly occurred at consecutive G or C nucleotides. Previous studies showed that G was most likely to react with formaldehyde and *N*^2^-hydroxymethyl-dG was the primary DNA adduct formed in cells following formaldehyde exposure [49].

Given the extensive genetic variation observed in the evolved strains, 16 genes that were commonly mutated in at least three of the four evolved strains were selected for further investigation. In total, 29 mutations were identified in these genes and 26 mutations were individually introduced into the starting strain FM-1 (Table 1, Supplementary Fig. 4 and Supplementary Table 3). Three mutations could not be successfully introduced despite several rounds of trials, including *cgl0752*^C929A^, *cgl0754*^391−411del^ (nucleotides 391–411 were deleted), and *cgl0754*^574−579del^ (nucleotides 574–579 were deleted). The gene *cgl0752* encodes *S*-adenosyl-l-homocysteine hydrolase that catalyzes the conversion of *S-*adenosyl-l-homocysteine to l-homocysteine, a precursor of l-methionine biosynthesis [50]. Although introduction of *cgl0752*^C929A^ failed, another mutation *cgl0752*^G761T^ was successfully introduced into strain FM-1. *cgl0754* encodes a response regulator (MtrA) of the two-component signal transduction system MtrAB, which participates in cell wall metabolism and osmostress response [51,52]. The 26 recombinants carrying the individual mutations were further evaluated for formaldehyde tolerance by assessing their growth on CGXII solid medium supplemented with 1.0 mM formaldehyde and 10 g/L glucose. Growth on medium without formaldehyde served as a control. All mutants exhibited growth patterns similar to the parental strain FM-1 in the absence of formaldehyde, indicating that the introduced mutation had no negative effect on growth (Supplementary Fig. 5). Among these, three mutants, *cgl1199*^1015−1032del^ (nucleotides 1015–1032 were deleted), *cgl1590*^535insC^ (a C was inserted at nucleotide 535), and *cgl1590*^750insG^ (a G was inserted at nucleotide 750), exhibited apparent growth advantages under formaldehyde stress (Fig. 2B). Subsequently, the growth of these three mutants was re-examined in CGXII liquid medium containing 0.8 mM formaldehyde or 0 mM formaldehyde. No growth differences were observed among the mutants and FM-1 under non-stress conditions (Supplementary Fig. 6). Under formaldehyde stress, growth analysis revealed that the *cgl1590*^750insG^ mutant exhibited a significantly shorter lag phase compared to FM-1, and the other two mutants also exhibited a modest but consistent improvement in growth under formaldehyde stress. These results confirmed the beneficial effects of three mutants on formaldehyde tolerance. Furthermore, the combinatorial mutant carrying both *cgl1590*^750insG^ and *cgl1199*^1015−1032del^ mutations exhibited enhanced formaldehyde tolerance, reflecting a synergistic effect between these two mutations (Fig. 2C). Meanwhile, glucose utilization of the evolved and mutant strains was assessed. FM-3 exhibited the fastest glucose consumption, the mutant carrying both *cgl1590*^750insG^ and *cgl1199*^1015−1032del^ mutations showed an intermediate rate, and FM-1 exhibited the slowest consumption (Supplementary Fig. 7). We further evaluated the effects of these mutations on methanol tolerance. However, likely owing to the distinct cytotoxic mechanisms of formaldehyde and methanol, these mutations did not confer any enhancement in methanol tolerance (Supplementary Fig. 8).

**Table 1:** Mutations of evolved strains identified by genome sequencing.

Gene ID	Gene name	Gene product	Nucleotide alteration	Amino acid change	Strain
*cgl0978*	*tdcB*	Threonine dehydratase	686insG	Frameshift mutation	FM-2
			G835T	A279S	FM-4 and FM-5
			G388T	A130S	FM-4
*cgl2495*	–	3-oxoacyl-(acyl-carrier-protein) synthase	G6160T	A2054S	FM-3, FM-4 and FM-5
			G6163T	A2055S	FM-4 and FM-5
*cgl0194*	–	Hypothetical protein	G971A	R324H	FM-2 and FM-3
			T506A	I169N	FM-4 and FM-5
*cgl1070*	–	ADP-ribose pyrophosphatase	T347A	V116D	FM-2
			T98C	V33A	FM-4 and FM-5
*cgl2303*	–	maltose alpha-*d*-glucosyltransferase	G779T	G260V	FM-4 and FM-5
			G624T	M208C	FM-2 and FM-3
*cgl1590*	–	Putative gluconeogenesis factor	535insC	Frameshift mutation	FM-2 and FM-3
			750insC	Frameshift mutation	FM-5
*cgl0279*	*whiB*	Transcriptional regulator WhiB	323insG	Frameshift mutation	FM-2, FM-3 and FM-5
*cgl1199*	rho	Transcription termination factor	1015-1032del	Frameshift mutation	FM-2, FM-3, FM-4 and FM-5
			T1226A	A409N	FM-4 and FM-5
*cgl2030*	–	Predicted ATPase with chaperone activity	C535T	P179S	FM-2, FM-3, FM-4 and FM-5
*cgl3085*	*pcnA*	tRNA nucleotidyltransferase/poly(A) polymerase	C1277A	A426D	FM-2, FM-3, FM-4 and FM-5
*cgl1369*	*uvrB*	Helicase subunit of the DNA excision repair complex	C648A	F216L	FM-4 and FM-5
			A1198T	T400S	FM-4 and FM-5
			G544T	V182F	FM-2 and FM-3
*cgl2482*	*glsK*	Glutaminase	A526T	T176S	FM-2 and FM-3
			G795T	V265V	FM-4 and FM-5
*cgl1373*	–	Hypothetical protein	2278insC	Frameshift mutation	FM-2, FM-3, FM-4 and FM-5
*cgl0752*	*sahH*	*S*-adenosylhomocysteine hydrolase	G761T	G254V	FM-2 and FM-3
			C929A	A310D	FM-4 and FM-5
*cgl0942*	*prsA*	ribose-phosphate pyrophosphokinase	G443T	G148V	FM-2, FM-3, FM-4 and FM-5
*cgl0754*	*mtrA*	DNA-binding response regulator MtrA	574-579del	Frameshift mutation	FM-2
			391-411del	Frameshift mutation	FM-4 and FM-5

The gene *cgl1199* encodes a transcription termination factor Rho that plays a central role in FMN riboswitch-mediated gene expression control [53] and pyrimidine *de novo* biosynthesis [54]. Disruption of *cgl1199* increased the transcription elongation efficiency under FMN rich conditions [53]. Structurally, Rho contains a non-conserved N-terminal domain (NTD) and a conserved C-terminal domain (CTD), which harbors the secondary binding site motifs (Q- and R-loops) critical for ATP-dependent RNA translocation and subunit interaction [[55], [56], [57]]. The *cgl1199*^1015−1032del^ mutation led to deletion of six amino acid residues (RNRRGR) from the position 339 to 344, which may affect the physiological function of Rho in transcription regulation. A predicted structure of Cgl1199^339−344del^ mutant, generated using Alphafold3 (https://alphafoldserver.com/) [45], showed that the six-residue deletion induced local structural changes distant from the CTD (Supplementary Fig. 9A and 9B), suggesting that direct interference with cofactor or substrate binding is unlikely. Cgl1590 is annotated by the UniProt database as a putative gluconeogenesis factor, which was required for morphogenesis under gluconeogenic growth conditions [58]. However, the function of Cgl1590 has not been experimentally characterized.

### 3.3 Effects of formaldehyde stress on the transcript profiles of the starting and evolved strains

**Fig. 3:**
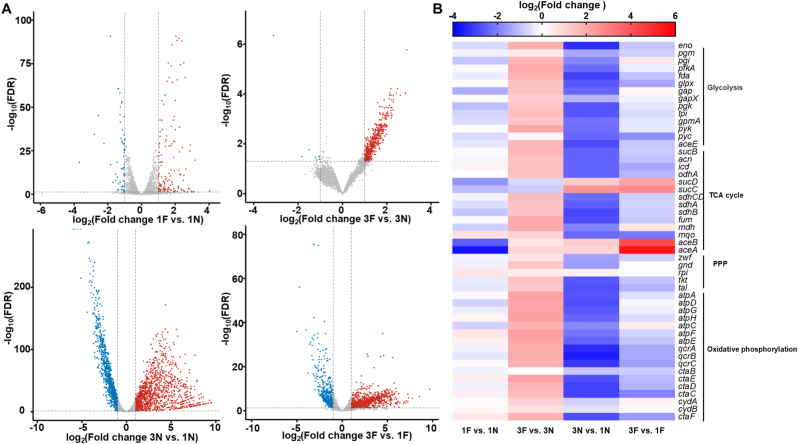
Transcriptome analysis of FM-3 and FM-1 cultivated with or without formaldehyde stress. (A) Volcano plots of differential transcription levels in 1F vs. 1N, 3F vs. 3N, 3N vs. 1N, and 3F vs. 1F. (B) Changes in mRNA levels of genes involved in central metabolism and the respiratory chain between FM-3 and FM-1. Only significant changes (log_2_(fold change) ≥1 or ≤ −1, FDR≤0.05) are shown. Upregulated and downregulated genes are indicated with red and blue, respectively. 1F, FM-1 cultivated with formaldehyde stress. 1N, FM-1 cultivated without formaldehyde stress. 3F, FM-3 cultivated with formaldehyde stress. 3N, FM-3 cultivated without formaldehyde stress.

To investigate the cellular response to formaldehyde stress, strains FM-1 and FM-3 were cultivated in CGXII minimal medium supplemented with 10 g/L glucose, with or without 0.8 mM formaldehyde. Cells at the middle exponential phase were harvested for transcriptome analysis. Three biological replicates were conducted. Pearson's correlation coefficient test and principal component analysis (PCA) showed high accuracy and reliable repeatability of the experimental methods (Supplementary Fig. 10A and 10B). Genes were classified as differentially expressed with a log_2_(Fold change) ≥1 or ≤ −1 and a false discovery rate (FDR) ≤0.05. By comparing the transcriptomes of starting strain FM-1 cultivated with or without formaldehyde, the response to formaldehyde stress was investigated in the genetic context of no formaldehyde dissimilation pathway. Exposing FM-1 to formaldehyde stress resulted in a total of 213 differentially expressed genes with statistical significance compared to non-stress condition. Among them, 144 and 69 genes were upregulated and downregulated, respectively (Fig. 3A and Supplementary Table 4). These differentially expressed genes were classified into 21 cellular processes according to KEGG_small_class annotation. A large proportion of genes fell in the functional categories of carbohydrate metabolism, amino acid metabolism, and membrane transport (Supplementary Fig. 11). Specifically, many genes involved in the glycolysis pathway (such as *gap*, encoding glyceraldehyde-3-phosphate dehydrogenase, and *pgk*, encoding 3-phosphoglycerate kinase) and tricarboxylic acid (TCA) cycle (such as *sucC* and *sucD*, encoding succinyl-CoA synthetase subunits) were downregulated when strain FM-1 was subjected to formaldehyde stress (Fig. 3B). Meanwhile, the expression levels of glyoxylate shunt genes *aceA* (isocitrate lyase) and *aceB* (malate synthase) were significantly downregulated by 11.88 and 4.89-fold, respectively. Regarding amino acid metabolism, most genes involved in biosynthesis of amino acids such as l-glutamate, l-tryptophan, l-arginine, l-cysteine, andl-leucine, were upregulated (Supplementary Table 4). However, the mRNA level of *metE*, encoding l-methionine synthase responsible for the formation of l-methionine from homocysteine [59], was significantly downregulated by 7.02-fold. Additionally, the *dnaE2* gene, encoding a DNA polymerase III alpha subunit involved in translesion DNA synthesis [60], displayed a 3.8-fold increase in presence of formaldehyde, suggesting a role in the repair of formaldehyde-induced DNA damage.

The evolved strain FM-3 exhibited a markedly enhanced transcriptional response to formaldehyde stress. A total of 480 genes showed statistically significant expression differences compared to no stress condition (Fig. 3A and Supplementary Table 4), over twice the number observed in the starting strain FM-1 (213 differentially expressed genes), suggesting a dramatic response of the evolved strain FM-3 to formaldehyde. These 480 genes were classified into 29 cellular processes based on KEGG_small_class annotation (Supplementary Fig. 12). Notably, only 10 genes were significantly downregulated, including those encoding 5′-nucleotidase/2′,3′-cyclic phosphodiesterase and related esterase, ABC-type transporter, and hypothetical membrane protein. In contrast, genes involved in oxidative phosphorylation and central metabolism such as TCA cycle, glycolysis, and pentose phosphate pathway (PPP) were almost upregulated (Fig. 3B). These findings demonstrate that the evolved strain FM-3 has developed a distinct transcriptional response mechanism to formaldehyde stress compared to the parental strain FM-1.

Next, we compared the differences in transcripts between the evolved strain FM-3 and the starting strain FM-1. In the absence of formaldehyde, over 63 % of the total *C. glutamicum* genome (1936 of total 3099 genes) of the evolved strain FM-3 showed significantly different expression levels, with 1077 and 859 genes upregulated and downregulated, respectively (Fig. 3A and Supplementary Table 4). Most of the genes involved in the functional category of replication and repair, such as base excision repair genes (*ung*, *mutY*, and *nth*) and homologous recombination genes (*ruvA*, *ruvB*, *ruvC*, *recF*, and *recR*), were upregulated based on KEGG_small_class annotation (Supplementary Fig. 13), suggesting enhanced DNA repair capacity in FM-3, possibly as an adaptive response developed during ALE to cope with formaldehyde-induced genotoxicity. Conversely, a lot of genes related to membrane transport, translation, energy metabolism, carbohydrate metabolism, folding, sorting and degradation, and nucleotide metabolism were downregulated. Specifically, pathways such as the oxidative phosphorylation, glycolysis pathway, PPP, pyruvate metabolism, and part of TCA cycle were downregulated, except for *sucC* and *sucD* (succinyl-CoA synthetase subunits), as well as *aceA* and *aceB* (key enzymes in the glyoxylate shunt) (Fig. 3B). The downregulation of central metabolism and energy metabolism may explain the reduced biomass of FM-3 under non-stress conditions (Fig. 1E). Whole-genome sequencing revealed that FM-3 harbors mutations in genes spanning diverse functional categories, including cell division, amino acid metabolism, transcriptional regulation, and the phosphotransferase system. Among these, mutations in phosphoenolpyruvate–protein phosphotransferase (*cgl1933*^T1112C^, *ptsI*) and pyruvate kinase (*cgl2089*^*T695A*C^, *pyk*), which are directly involved in central carbon metabolism, may have a more direct impact on metabolic flux distribution, thereby potentially contributing to the observed transcriptional changes of central metabolic genes.

To explore the transcriptional basis for the improved formaldehyde tolerance of strain FM-3, we compared the gene expression profiles of FM-3 and FM-1 under formaldehyde stress conditions. A total of 1243 genes were differentially expressed, including 784 and 459 upregulated and downregulated genes in FM-3 relative to FM-1 (Fig. 3A and Supplementary Table 4). Most genes associated with membrane transport, translation, energy metabolism, carbohydrate metabolism and nucleotide metabolism were downregulated based on KEGG_small_class annotation (Supplementary Fig. 14). For instance, genes in glycolysis, oxidative phosphorylation, TCA cycle were suppressed, apart from *sucC* and *sucD* that were upregulated by 5.47-fold and 3.52-fold, respectively (Fig. 3B). Some genes involved in amino acid synthesis, such as l-glutamate, l-tryptophan, l-arginine, l-leucine, l-threonine, l-glutamine, l-serine, l-glycine, l-proline, and l-tyrosine, were also downregulated (Supplementary Table 4). Interestingly, the expression of *metE*, encoding l-methionine synthase, was significantly upregulated by 11.61-fold. Meanwhile, most genes involved in DNA damage repair were upregulated. Base excision repair genes *ung* (*cgl1324*), *mutY* (*cgl2671*), *nth* (*cgl0293*), and *mutM2* (*cgl3000*) were upregulated by 1.22-, 2.12-, 1.50-, and 3.43-fold, respectively, and homologous recombination genes *ruvA* (*cgl1661*) and *ruvB* (*cgl1660*) were upregulated by 1.86- and 1.21-fold, respectively. In addition, the mRNA level of *dnaE2* involved in translesion DNA synthesis was upregulated by 4.49-fold. These regulation events may facilitate faster growth and higher tolerance of strain FM-3 under formaldehyde stress (Fig. 1C). In addition, we examined the transcriptional changes of shared mutated genes in the evolved strains. Comparative transcriptomic analysis revealed that most shared mutated genes exhibited differential expression in FM-3 relative to FM-1 under both formaldehyde stress and non-stress conditions (Supplementary Table 5). Notably, the mRNA level of *cgl1590* was significantly downregulated in FM-3 compared to FM-1 under formaldehyde-free condition. Similarly, *cgl1199* transcripts were consistently downregulated in FM-3 compared to FM-1 regardless of formaldehyde treatment.

### 3.4 Proteomic analysis of the starting and evolved strains

**Fig. 4:**
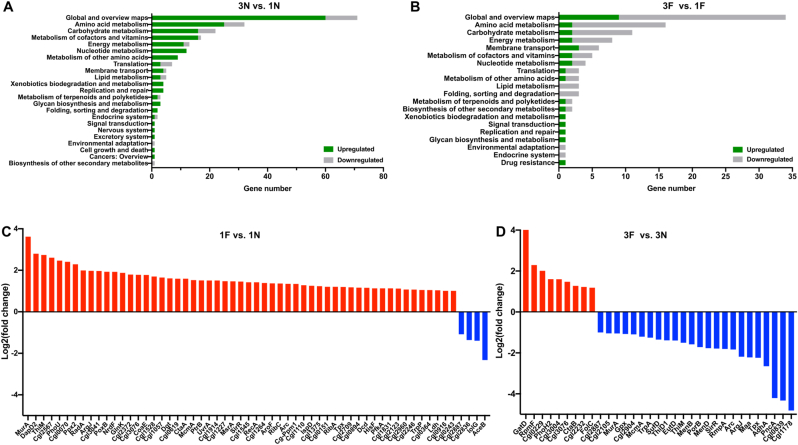
Comparison of proteomes between FM-3 and FM-1 cultivated with formaldehyde or without formaldehyde stress. Functional classification of transcriptome differences based on KEGG_small_class annotation in 3N vs. 1N (A), 3F vs. 1F (B). Proteins with differentially expression in 1F vs. 1N (C) and 3F vs. 3N (D). Only significant changes (log_2_(fold change) ≥1 or ≤ −1, FDR≤0.05) are shown. Proteins exhibiting increased or decreased abundance are highlighted in red and blue, respectively. 1F, FM-1 cultivated with formaldehyde stress. 1N, FM-1 cultivated without formaldehyde stress. 3F, FM-3 cultivated with formaldehyde stress. 3N, FM-3 cultivated without formaldehyde stress.

To investigate potential proteome remodeling during the adaptive evolution, proteomic analysis was performed on strains FM-1 and FM-3 cultivated in CGXII minimal medium containing 10 g/L glucose, with or without 0.8 mM formaldehyde. Three independent biological replicates were conducted. Over 1000 proteins were detected in each sample. Pearson's correlation coefficient test and PCA of the proteomic analysis showed a high accuracy and statistically reliable repeatability of the experiments (Supplementary Fig. 15A and 15B). In the absence of formaldehyde, a total of 207 proteins exhibited statistically significant differences in abundance in the evolved strain FM-3 compared to FM-1. Among them, the abundance of 165 proteins was increased, while 42 proteins showed decreased abundance. Enzymes involved in nucleotide metabolism, replication and repair, glycan biosynthesis and metabolism, and part of energy metabolism pathways showed increased abundance based on KEGG_small_class annotation (Fig. 4A and Supplementary Table 6). In particular, several enzymes involved in l-methionine metabolism showed increased abundance, including MetB (Cgl2446, cystathionine-γ-synthase), MetX (Cgl0652, homoserine *O*-acetyltransferase), MetE, MetY (Cgl0653, *O*-acetyl-l-homoserine sulfhydrylase), CysD (Cgl2815, sulfate adenylyltransferase subunit 2), CysK (Cgl2562, cysteine synthase), which were increased by 4.69-, 1.65-, 1.55-, 1.43-, 4.35-, and 2.10-fold, respectively. When integrated with the transcriptomic analysis, we observed that ribosomal protein RpsS (Cgl0511, ribosomal protein S19) and RpsH (Cgl0537, ribosomal protein S8) showed consistent downregulation at both the transcriptional and protein levels, suggesting a reduction in translational capacity, which may be associated with the decreased biomass of FM-3 under non-stress conditions. Moreover, both AceA and AceB displayed concordant upregulation at the mRNA and protein levels. When cells were exposed to formaldehyde stress, 108 proteins showed significant differences in abundance between FM-3 and FM-1. Among them, the abundance of 30 proteins increased, whereas 78 proteins showed decreased abundance. Proteins involved in amino acid metabolism and energy metabolism showed decreased abundance (Fig. 4B and Supplementary Table 6). However, increased abundance was observed for MetE, MurE (UDP-*N*-acetylmuramoyl-l-alanyl-d-glutamate-2,6-diaminopimelate ligase, cell-wall peptidoglycan biosynthesis), and LigA (DNA ligase, DNA replication and damage repair).

We further analyzed proteomic changes in FM-1 and FM-3 in response to formaldehyde stress. In FM-1, 59 proteins exhibited significant differences in abundance under formaldehyde stress compared to the non-stress condition. Among them, 55 proteins showed increased abundance, and 4 proteins showed decreased abundance (Fig. 4C). For example, the abundances of MurA (Cgl0352, UDP-*N*-acetylglucosamine enolpyruvyl transferase) and Cg-Ppm1 (Cgl1478, polyprenyl monophosphomannose synthase) [61], both involved in cell wall biogenesis, increased by 11.29- and 1.53-fold, respectively. Additionally, DNA damage repair proteins UvrA (Cgl1376) and RecA (Cgl1955) exhibited increased abundance by 1.85- and 1.68-fold, respectively, which was consistent with the upregulation of DNA replication and repair genes (*ung*, *mutY*, *nth*, *ruvA*, *ruvB*, *ruvC*, *recF*, *recR*) observed in the transcriptome. We further found that AceB and AroF (Cgl0990, 3-deoxy-d-arabino-heptulosonate 7-phosphate (DAHP) synthase), involved in central carbon and aromatic amino acid metabolism, displayed consistent upregulation at the transcriptional and protein levels under formaldehyde stress. For the evolved strain FM-3, a total of 33 proteins showed statistically significant differences in abundance when cells were cultivated with formaldehyde stress compared to the non-stress condition, with 9 proteins showing increased abundance and 24 showing decreased abundance (Fig. 4D). Notably, GatD (lipid II isoglutaminyl synthase), which catalyzes the formation of alpha-d-isoglutamine in the cell wall lipid II stem peptide [62], showed a dramatic 15.21-fold increase in abundance. In contrast, Cgl0839 (homoserine acetyltransferase), which is involved in the l-methionine cycle and catalyzes the conversion of l-homoserine to *O*-acetyl-l-homoserine, was decreased by 19.06-fold in abundance. Furthermore, we analyzed protein abundance changes of shared mutated genes in the evolved strains (Supplementary Table 5). Under non-stress conditions, the protein abundance of Cgl2482 (glutaminase) in FM-3 was increased by 1.20-fold, whereas that of Cgl0752 (*S*-adenosylhomocysteine hydrolase) was decreased by 1.68-fold compared to FM-1. In addition, Cgl0942 (ribose-phosphate pyrophosphokinase) showed a 1.49-fold decrease in protein abundance in FM-3 relative to FM-1 under formaldehyde stress. By contrast, Cgl1590 and Cgl1199 displayed no significant changes in protein abundance, which differed from their transcriptional profiles. Overall, cell wall synthesis and DNA damage repair are crucial for cellular formaldehyde tolerance, and the l-methionine cycle appears to be closely associated with formaldehyde resistance, though the precise mechanisms remain to be elucidated.

### 3.5 Functional analysis of Cgl1590 and its truncated variant

**Fig. 5:**
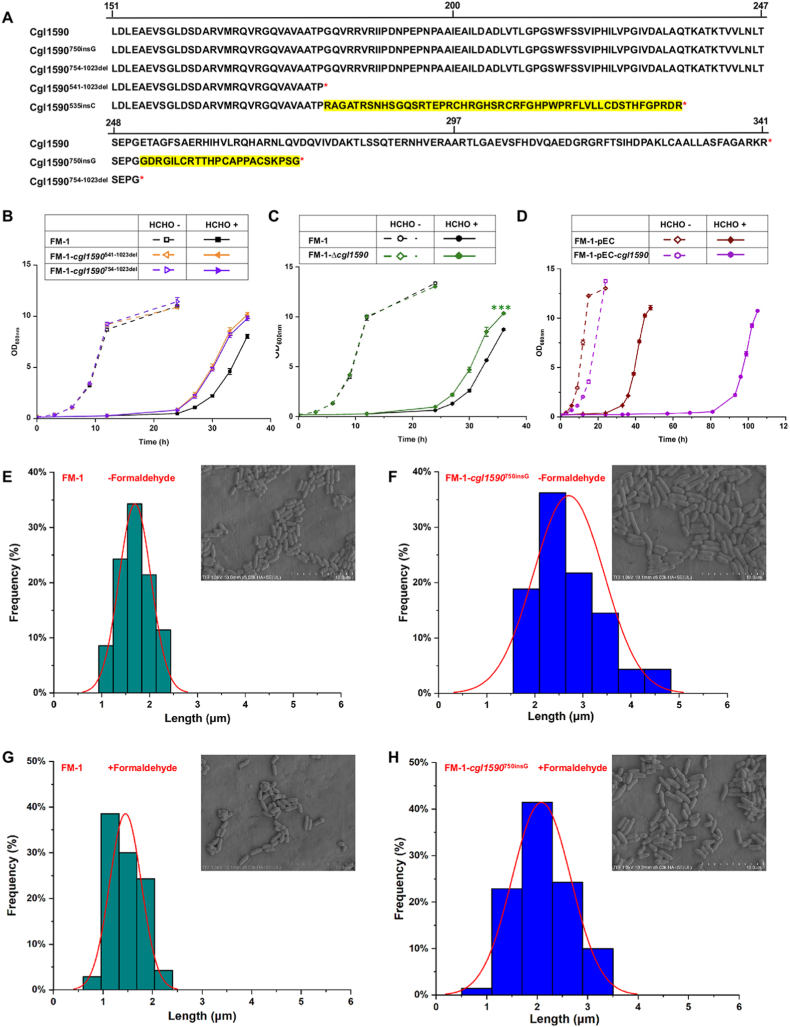
Effects of Cgl1590 mutations on formaldehyde tolerance. (A) Amino acid sequence alignment between Cgl1590 and its derivatives. (B) Effects of *cgl1590* truncation on formaldehyde tolerance. (C) Effects of *cgl1590* knock-out on formaldehyde tolerance. (D) Effects of *cgl1590* overexpression on formaldehyde tolerance. Cells were treated with 0.8 mM formaldehyde as stress condition. Values and error bars reflect the mean ± s.d. of three biological replicates (N = 3). Analysis of cell length and morphology of FM-1 without formaldehyde stress (E), FM-1 containing *cgl1590*^750insG^ mutation without formaldehyde stress (F), FM-1 with formaldehyde stress (G), and FM-1 strain containing *cgl1590*^750insG^ mutation with formaldehyde stress (H). All strains were grown in CGXII minimal medium supplemented with 10 g/L glucose, with or without 0.8 mM formaldehyde, and examined by SEM. Cell length was determined by measuring 70 cells of each strain and analyzed using ImageJ software. Statistical significance at 36 h between FM-1-△*cgl1590* and FM-1 was determined by two-tailed Student's *t*-test: ∗∗∗P < 0.001.

Two *cgl1590* mutations led to the improvement in formaldehyde tolerance of the strain, thus the function of *cgl1590* was further investigated. The length of *cgl1590* gene was 1026 bp, encoding 341 amino acid residues. The *cgl1590*^535insC^ and *cgl1590*^750insG^ mutations resulted in a frameshift at amino acid position 181 and 252, respectively, resulting in premature stop codons at positions 230 and 274 (the partial amino acid sequences were compared in Fig. 5A). The protein truncation caused by frameshift mutations might lead to the loss-of-function of Cgl1590. To test this hypothesis, two truncated mutations, *cgl1590*^541−1023del^ mutation (nucleotides 541–1023 corresponding to the amino acid sequence of 181–341 were deleted) and *cgl1590*^754−1023del^ mutation (nucleotides 754–1023 corresponding to the amino acid sequence of 252–341 were deleted), were introduced to the chromosome of strain FM-1, respectively, and designated as FM-1-*cgl1590*^541−1023del^ and FM-1-*cgl1590*^754−1023del^. Another loss-of-function mutant was also constructed by knocking out the full-length *cgl1590* gene in strain FM-1 (designated as FM-1-Δ*cgl1590*). Then, growth of the strains was compared in CGXII minimal medium supplemented with 0.8 mM formaldehyde to characterize formaldehyde tolerance (Fig. 5B, C, and 5D). Two truncation strains (Fig. 5B) and the knocking-out strain (Fig. 5C) apparently grew better than the control strain FM-1 in presence of formaldehyde, confirming that the loss-of-function of *cgl1590* was beneficial to formaldehyde tolerance. However, their improvements remained lower than that of the FM-1-*cgl1590*^750insG^ mutant. Glucose utilization of the evolved and mutant strains was assessed. FM-3 exhibited the fastest glucose consumption, the mutant carrying both *cgl1590*^750insG^ and *cgl1199*^1015−1032del^ mutations showed an intermediate rate, and FM-1 exhibited the slowest consumption (Supplementary Fig. 7). Meanwhile, IPTG-inducible overexpression of *cgl1590* showed a negative effect on cell growth both in the presence and absence of formaldehyde (Fig. 5D), which gives a contrary phenotype to the mutants *cgl1590*^535insC^ and *cgl1590*^750insG^. Taken together, the overexpression of Cgl1590 was harmful, but the partial or total functional loss of Cgl1590 by the frameshift mutation, truncation, or knockout was beneficial for formaldehyde tolerance. Protein homology analysis showed that Cgl1590 shares 56% sequence identity with CuvA (carbon metabolism and virulence-associated protein A) from *Mycobacterium tuberculosis* [63]. CuvA belongs to the UPF0052 uncharacterized protein family and contains a Rothman fold, a motif commonly found in dinucleotide-binding proteins [64]. In *Mycobacterium*, CuvA localizes to the growing cell pole, the site of peptidoglycan synthesis, and its deletion has been shown to affect nutrient uptake, cell wall morphology, and virulence [63]. Given the sequence similarity, we hypothesized that Cgl1590 of *C. glutamicum* might share a similar function in cell wall synthesis. Scanning electron microscope (SEM) was used to observe the morphology of FM-1 (Fig. 5E and G) and FM-1 containing *cgl1590*^750insG^ mutation (Fig. 5F and H) under both formaldehyde stress and non-stress conditions. The graphs clearly showed that the *cgl1590*^750insG^ mutant exhibited increased cell length and greater morphological heterogeneity compared to FM-1, regardless of the presence or absence of formaldehyde. Therefore, we propose that *cgl1590* is involved in the regulation of cell morphology, and that the frameshift mutation alters its physiological function. These findings suggest that *cgl1590* may serve as a promising genetic target for engineering improved cellular resistance to formaldehyde.

## 4 Discussion

C1-based biomanufacturing has received widespread attention in recent years. Formaldehyde is a crucial intermediate in C1 bioconversion processes. However, as a highly toxic substance, formaldehyde can limit cell growth and enzymatic reaction rates. Although several mechanisms conferring formaldehyde tolerance have been uncovered in different microorganisms [18,[21], [22], [23]], a comprehensive understanding of how cells globally respond to formaldehyde stress remains limited. In particular, the broader impact of formaldehyde exposure on cellular metabolism, gene regulation, and stress adaptation remains poorly understood. Thus, a systematic investigation integrating multi-omics approaches is essential to reveal the molecular basis of formaldehyde tolerance.

Previous studies in other microorganisms have identified genes contributing to formaldehyde tolerance. For instance, proline aminopeptidase PepP in *E. coli* is capable of cleaving thioproline-containing peptides, mitigating formaldehyde-induced aberrant peptides [23]. Zhu et al. demonstrated that combining DNA-protein cross-link protease (GCNA1) from *Caenorhabditis elegans* with PepP from *E. coli* effectively reduced formaldehyde-induced DNA and protein damage, significantly enhancing strain tolerance to 1.2 mM formaldehyde [65]. In addition, other studies have shown that enhancing reactive oxygen species (ROS) scavenging systems in *E. coli* or introducing non-native, eukaryote-featured membrane phospholipid composition such as phosphatidylcholine to remodel the bacterial cell membrane can also markedly improve formaldehyde tolerance [66,67]. In this study, we obtained three mutants that could tolerate 2.6 mM formaldehyde by ALE, and demonstrated that *cgl1199*^1015−1032del^, *cgl1590*^535insC^ and *cgl1590*^750insG^ mutations were beneficial for improved formaldehyde tolerance. The *cgl1199* mutation likely enhances tolerance by globally altering transcriptional regulation networks, while *cgl1590* frameshift mutations directly improve resistance through cell morphology changes. Further investigation of the functions of both Cgl1199 and Cgl1590 will not only elucidate their respective roles, but also provide deeper insights into the cellular response to formaldehyde stress. Meanwhile, we systematically analyzed the global cellular metabolic changes induced by formaldehyde using multi-omics approaches. We observed that many genes involved in DNA replication and repair, as well as in peptidoglycan biosynthesis, were significantly upregulated in strains FM-1 and FM-3 under formaldehyde stress. This indicates that the cells actively initiate repair mechanisms to counteract the severe damage to DNA and proteins caused by formaldehyde. Nevertheless, our integrated transcriptomic and proteomic analyses revealed that FM-3 has evolutionarily optimized its metabolic network by upregulating key glyoxylate cycle components. This potential reinforcement of metabolic flux through the glyoxylate shunt likely contributes to improved formaldehyde tolerance in FM-3. Meanwhile, SDS-PAGE and mass spectrometry analyses revealed a substantial increase in MetE abundance in the evolved strain FM-3 (Supplementary Fig. 16A). However, overexpression of MetE in the starting strain FM-1 did not confer improved formaldehyde tolerance (Supplementary Fig. 16B), suggesting that the elevated abundance of MetE may reflect adaptive changes accumulated during the ALE process.

In conclusion, ALE proved to be an effective strategy for enhancing the formaldehyde tolerance of *C. glutamicum*. Integrated multi-omics analyses revealed key metabolic and regulatory adaptations underlying the cellular response to formaldehyde stress. Furthermore, the identified genetic mutations offer valuable insights for the rational engineering of formaldehyde-tolerant strains and hold potential for future applications in C1 compound biotransformation.

## CRediT authorship contribution statement

**Liwen Fan:** Writing – review & editing, Writing – original draft, Visualization, Validation, Software, Methodology, Investigation, Funding acquisition, Formal analysis, Data curation, Conceptualization. **Qichen Cao:** Validation, Software, Methodology, Investigation, Data curation. **Zhihui Zhang:** Visualization, Validation, Methodology, Investigation, Data curation. **Xiaomeng Ni:** Visualization, Software, Methodology, Data curation. **Yu Lei:** Methodology, Investigation, Data curation. **Tuo Shi:** Validation, Methodology, Data curation. **Jiuzhou Chen:** Writing – original draft, Methodology, Formal analysis, Conceptualization. **Shengping Zhang:** Validation, Methodology, Data curation. **Wenjuan Zhou:** Project administration, Formal analysis, Data curation. **Yu Wang:** Writing – review & editing, Writing – original draft, Visualization, Resources, Project administration, Methodology, Investigation, Funding acquisition, Formal analysis, Data curation, Conceptualization. **Ping Zheng:** Writing – review & editing, Writing – original draft, Supervision, Resources, Project administration, Funding acquisition, Formal analysis, Data curation, Conceptualization. **Jibin Sun:** Validation, Supervision, Resources, Formal analysis, Data curation, Conceptualization.

## Declaration of competing interest

The authors declare that they have no known competing financial interests or personal relationships that could have appeared to influence the work reported in this paper. The author Yu Wang is an Editorial Board Member for Synthetic and Systems Biotechnology and was not involved in the editorial review or the decision to publish this article.

## Data Availability

All R scripts and input data used in this study have been uploaded and are available at https://github.com/YuWangLab/formaldehyde-tolerance.
