# Supplementary material for: Multi-omics analyses of evolved Corynebacterium glutamicum mutants reveal the molecular responses to formaldehyde stress
Source: Synth Syst Biotechnol. 2026 Feb 10;13:217–28. doi: 10.1016/j.synbio.2026.01.020 (PMC12914118; doi:10.1016/j.synbio.2026.01.020)
Supplement: Multimedia component 1 [file mmc1.docx]

**Supplementary information for**

**Multi-omics analyses of evolved *Corynebacterium glutamicum* mutants reveal the molecular responses to formaldehyde stress**

Liwen Fan,^a,b,1^ Qichen Cao,^a,b,1^ Zhihui Zhang,^a,^^b^ Xiaomeng Ni,^a,b^ Yu Lei,^a,b^ Tuo Shi,^a,b^ Jiuzhou Chen,^a,^^b^ Shengping Zhang,^a,b,c^ Wenjuan Zhou,^a,b^ Yu Wang,^a,b,d,*^ Ping Zheng^a,b,d,*^ and Jibin Sun^a,b,d^

^a^Key Laboratory of Engineering Biology for Low-carbon Manufacturing, Tianjin Institute of Industrial Biotechnology, Chinese Academy of Sciences, Tianjin 300308, China

^b^National Center of Technology Innovation for Synthetic Biology, Tianjin 300308, China

^c^College of Biotechnology, Tianjin University of Science and Technology, Tianjin, 300457, China.

^d^University of Chinese Academy of Sciences, Beijing 100049, China

^1^Co-first author with equal contribution

^*^Correspondence: Y. Wang (wang_y@tib.cas.cn) and P. Zheng (zheng_p@tib.cas.cn)


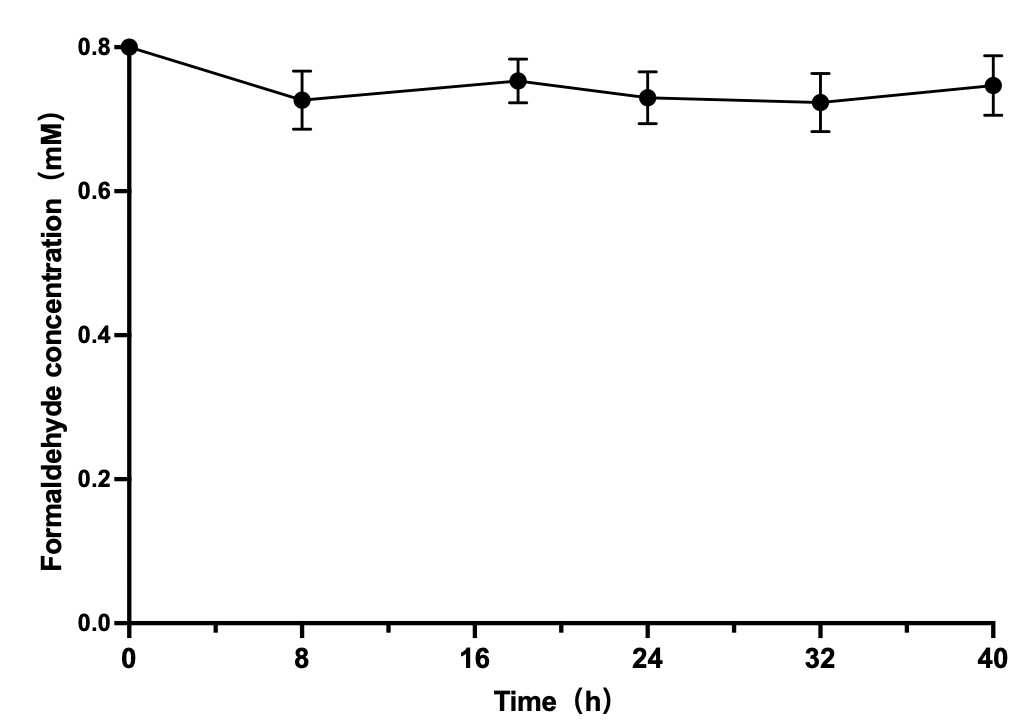


**Supplementary Fig. 1.** Formaldehyde concentration measurement in a blank shake flask containing 0.8 mM formaldehyde under sealed cultivation conditions (N=3).


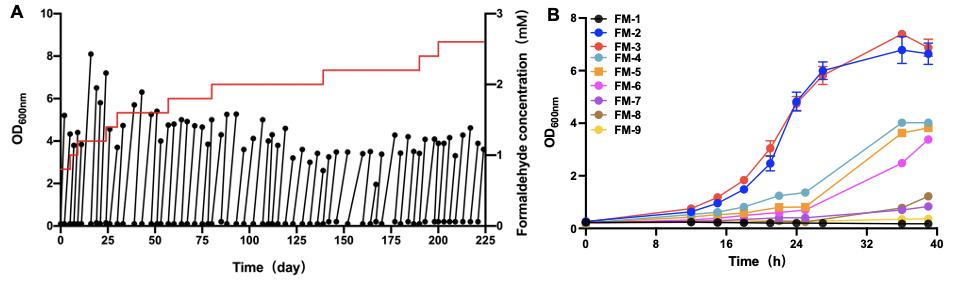


**Supplementary Fig. 2.** Improving the tolerance to formaldehyde via ALE. (A) ALE of culture-2 in CGXII minimal medium supplemented with different formaldehyde concentrations and 10 g/L glucose. (B) Growth curves of the evolved mutants isolated from culture-1 and culture-2 in CGXII minimal medium supplemented with 10 g/L glucose and 1.6 mM formaldehyde (N=3 or 1). **The sample size (N) of FM-1, FM-2, and FM-3 was 3, while** other mutants **had N=1**.


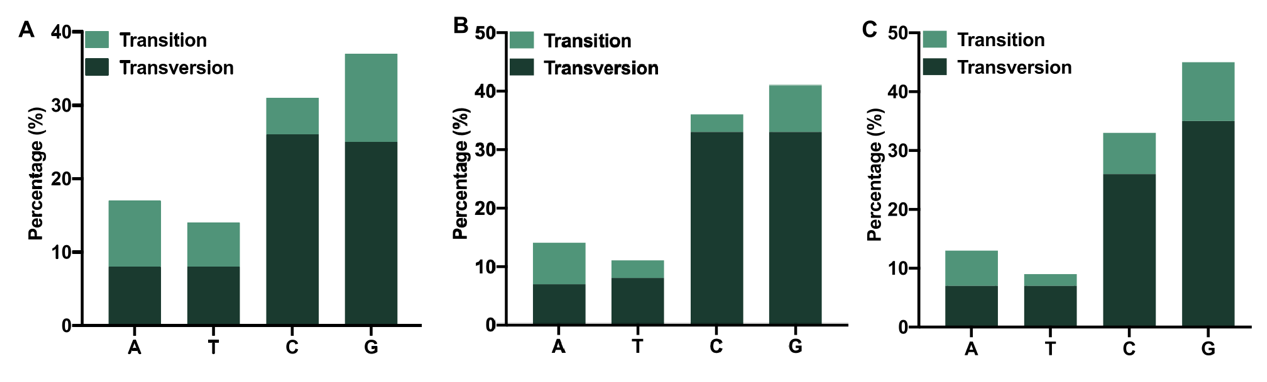


**Supplementary Fig. 3.** Analysis of the frequency of base mutation types in the evolved strains. Frequency of mutations of four bases in evolved strain FM-2(A), FM-4 (B), FM-5 (C).


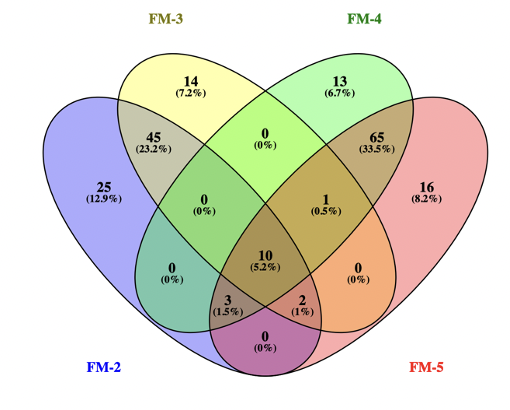


**Supplementary Fig. 4.** Venn diagram of mutant genes in the evolved strains.


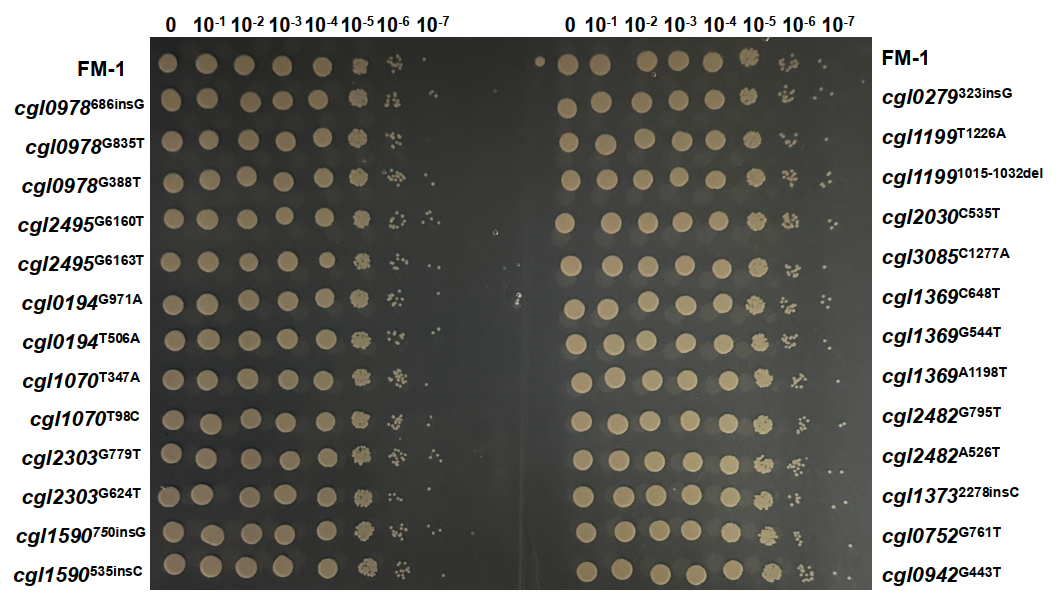


**Supplementary Fig. 5.** Growth of strain FM-1 and its derivatives harboring single-site mutations on CGXII minimal agar medium supplemented with 10 g/L glucose without formaldehyde.

*
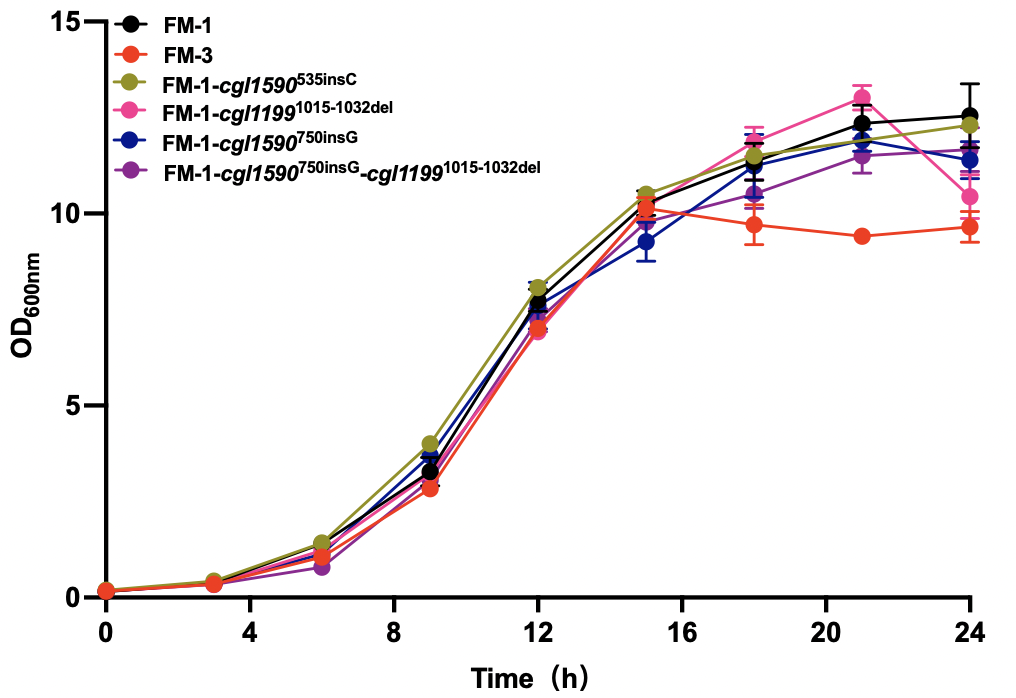
*

**Supplementary Fig. 6.** Growth curve of strain FM-1 and its derivatives in CGXII medium supplemented with 10 g/L glucose without formaldehyde. Values and error bars reflect the mean ± s.d. of three biological replicates (N = 3).


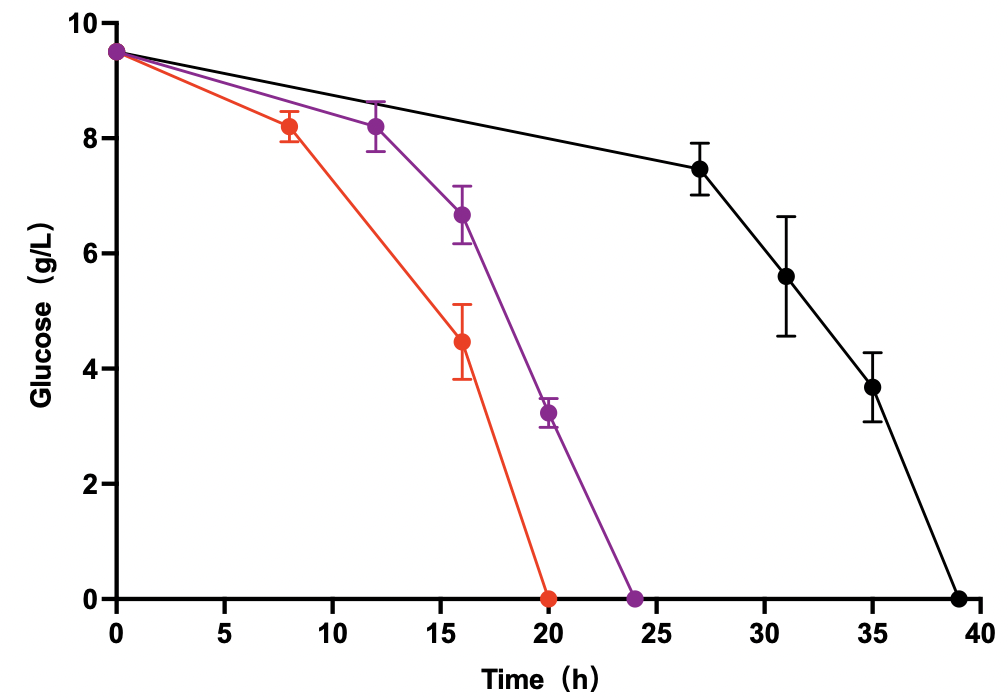


**Supplementary Fig. 7.** Glucose consumption profiles of the strain FM-1, FM-3 and FM-1-*cgl1590*^750insG^-*cgl1199*^1015-1032del^ were assessed in CGXII medium supplemented with 10 g/L glucose with 0.8 mM formaldehyde. Red represents FM-3, black represents FM-1, and purple represents FM-1-cgl1590750insG-cgl11991015-1032del. Values and error bars reflect the mean ± s.d. of three biological replicates (N = 3).


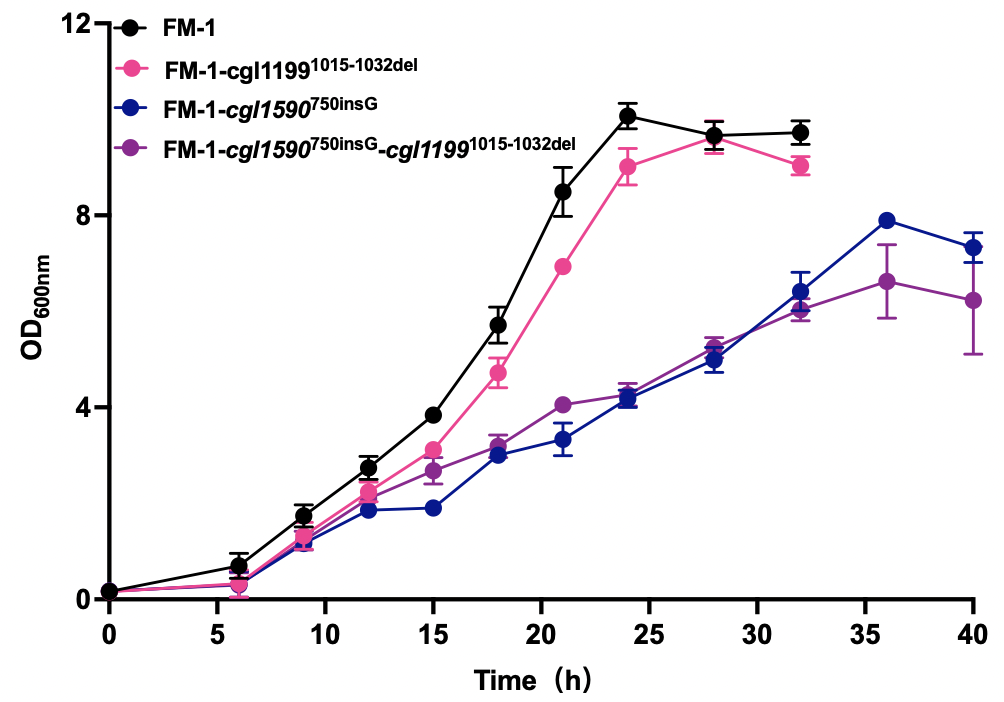


**Supplementary Fig. 8.** Growth curves of strain FM-1 and its derivatives in CGXII medium supplemented with 10 g/L glucose and 30 g/L methanol. Values and error bars reflect the mean ± s.d. of three biological replicates (N = 3).


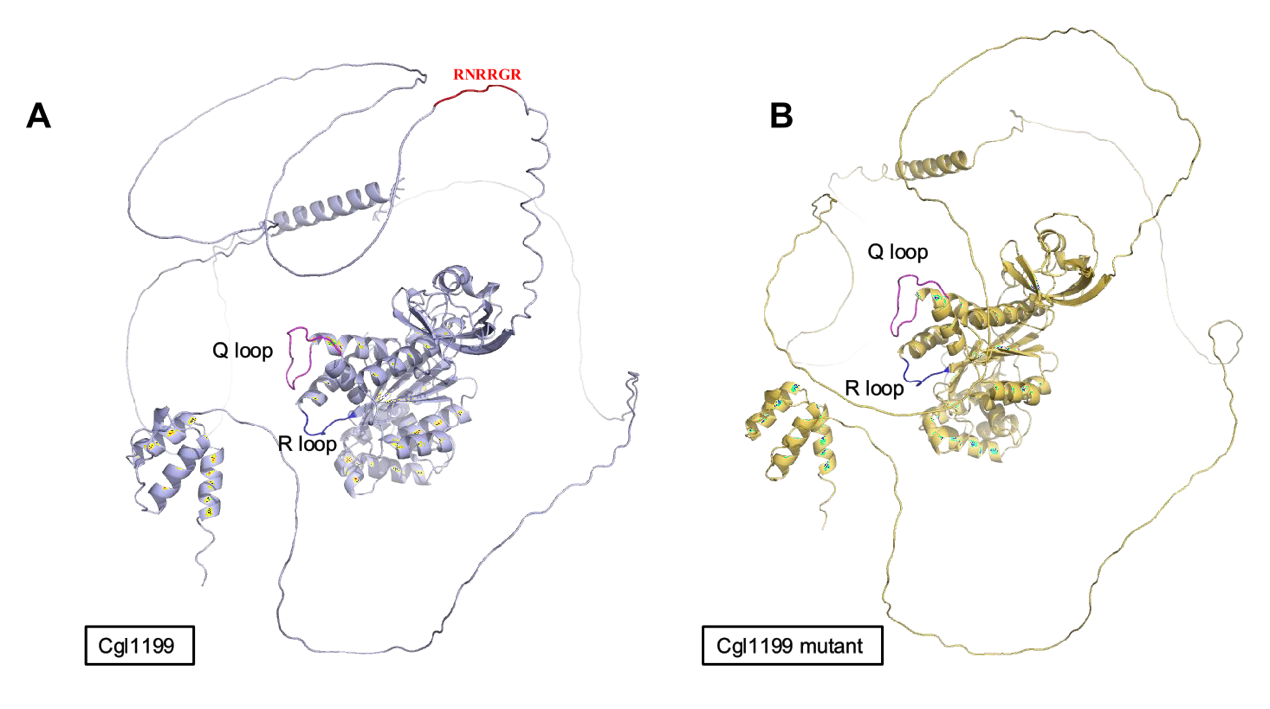


**Supplementary Fig.9.** **Structure prediction of Cgl1199 (A) and its mutant (B) using AlphaFold3**. The deleted six amino acid residues are indicated in red. The deleted amino acid residues caused changes in protein which were far from CTD.


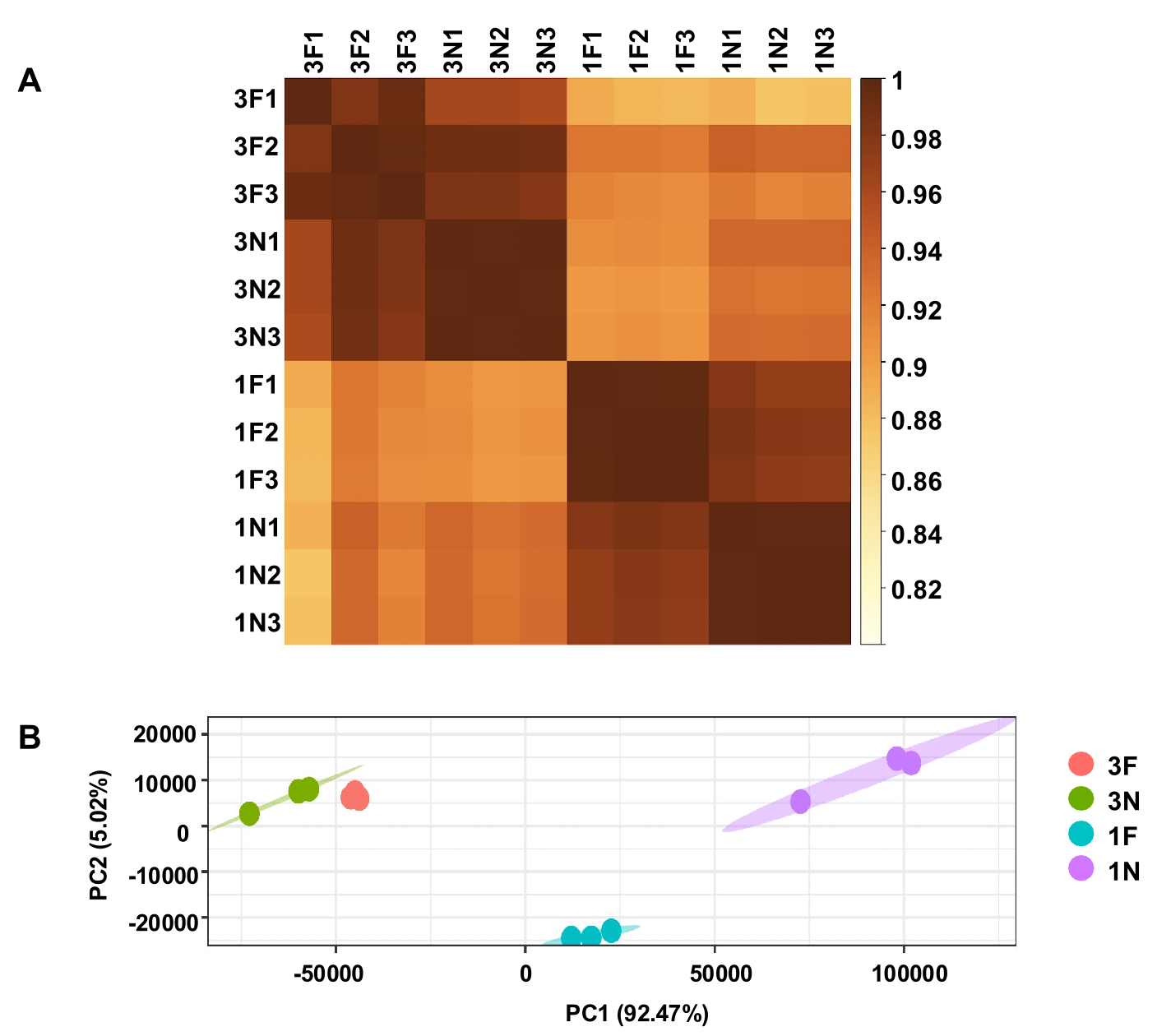


**Supplementary Fig. 10.** Evaluation of the accuracy and repeatability of transcriptome analysis. (A) Pearson’s correlation coefficient test. (B) Principal component analysis (PCA). 1F, FM-1 cultivated with formaldehyde stress. 1N, FM-1 cultivated without formaldehyde stress. 3F, FM-3 cultivated with formaldehyde stress. 3N, FM-3 cultivated without formaldehyde stress. Three biological replicates were performed.


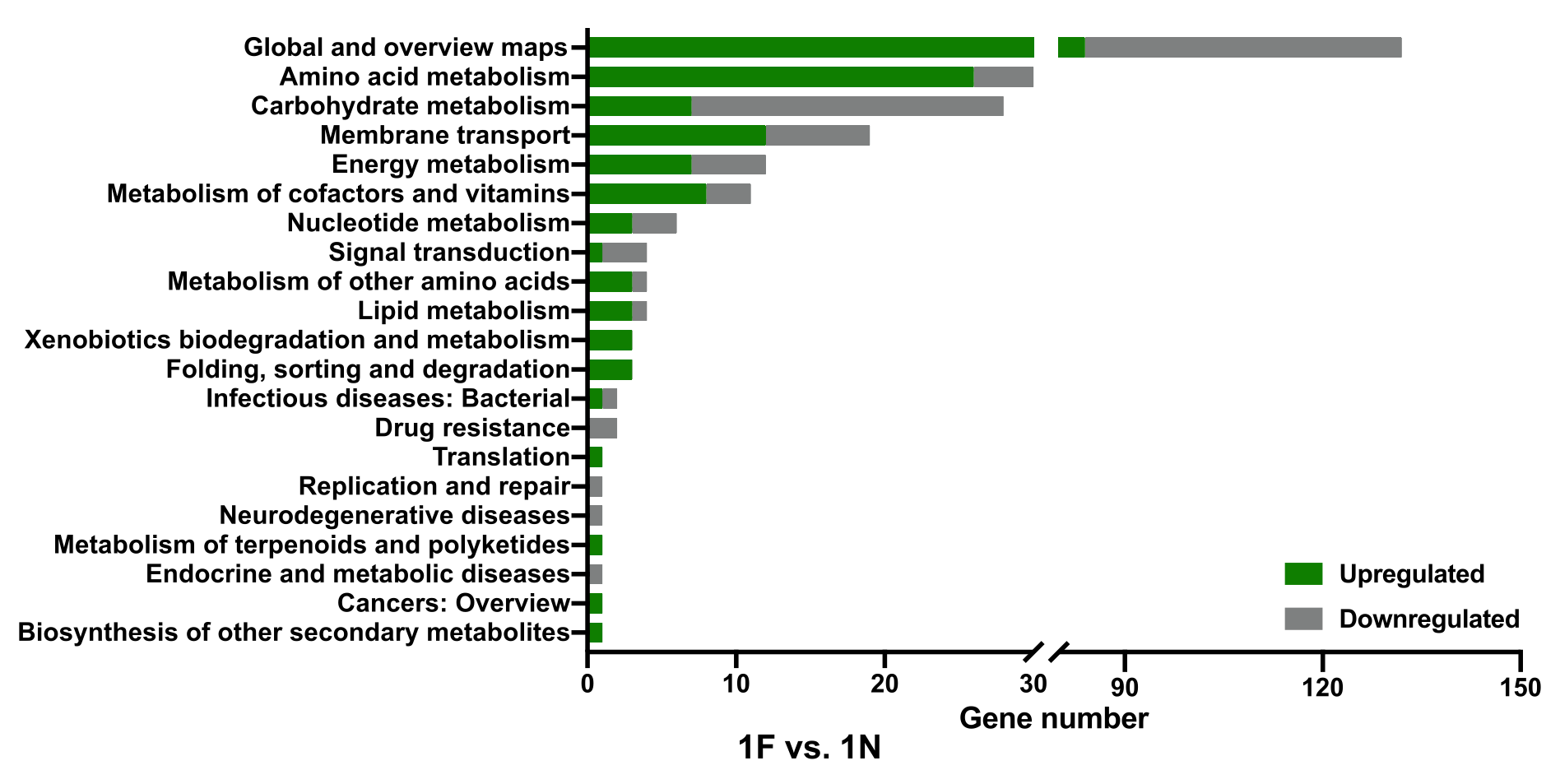
 **Supplementary Fig. 11.** Functional classification of transcriptome differences based on KEGG_small_class annotation in 1F vs. 1N. Upregulated and downregulated genes are indicated with green and gray, respectively. 1F, FM-1 cultivated with formaldehyde stress. 1N, FM-1 cultivated without formaldehyde stress. Three biological replicates were performed.


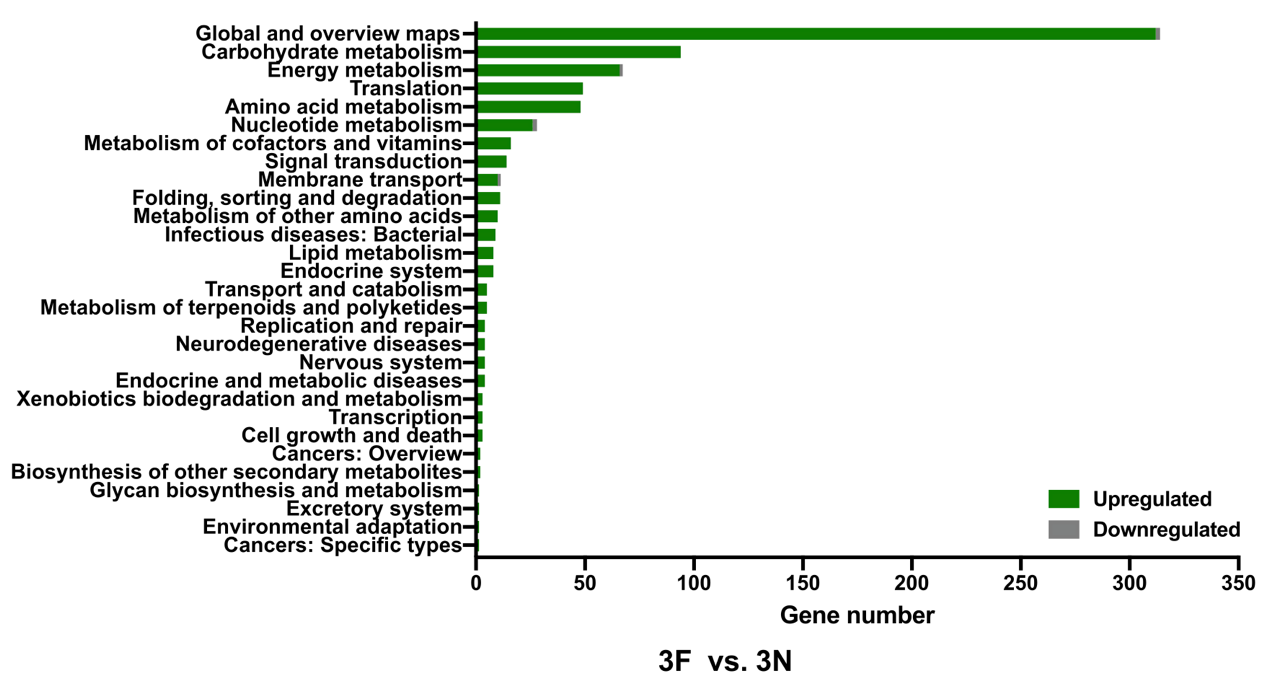


**Supplementary Fig. 12.** Functional classification of transcriptome differences based on KEGG_small_class annotation in 3F vs. 3N. Upregulated and downregulated genes are indicated with green and gray, respectively. 3F, FM-3 cultivated with formaldehyde stress. 3N, FM-3 cultivated without formaldehyde stress. Three biological replicates were performed.

**
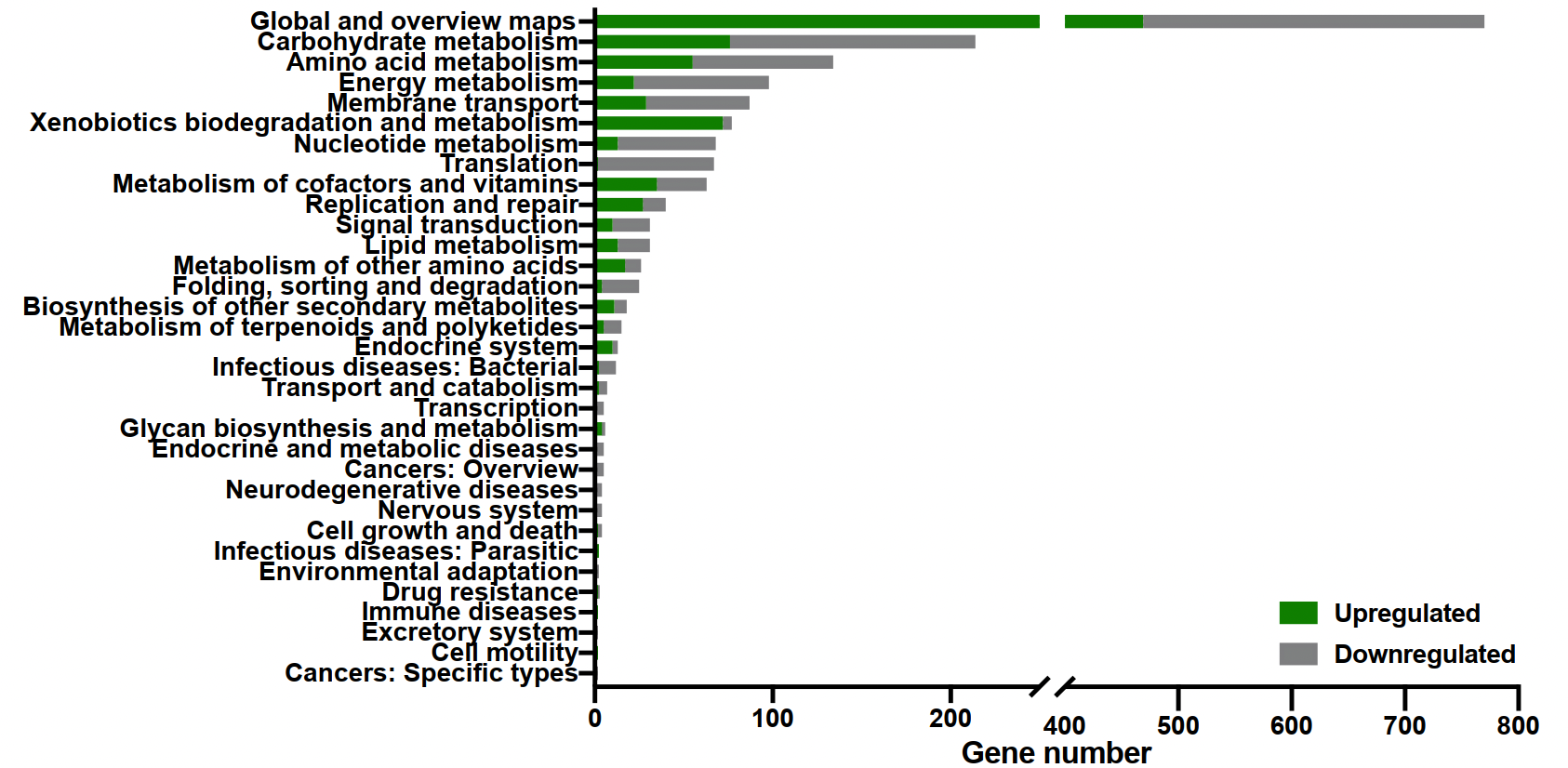
Supplementary Fig. 13.** Functional classification of transcriptome differences based on KEGG_small_class annotation in 3N vs. 1N. Upregulated and downregulated genes are indicated with green and gray, respectively. 1N, FM-1 cultivated without formaldehyde stress. 3N, FM-3 cultivated without formaldehyde stress. Three biological replicates were performed.

**
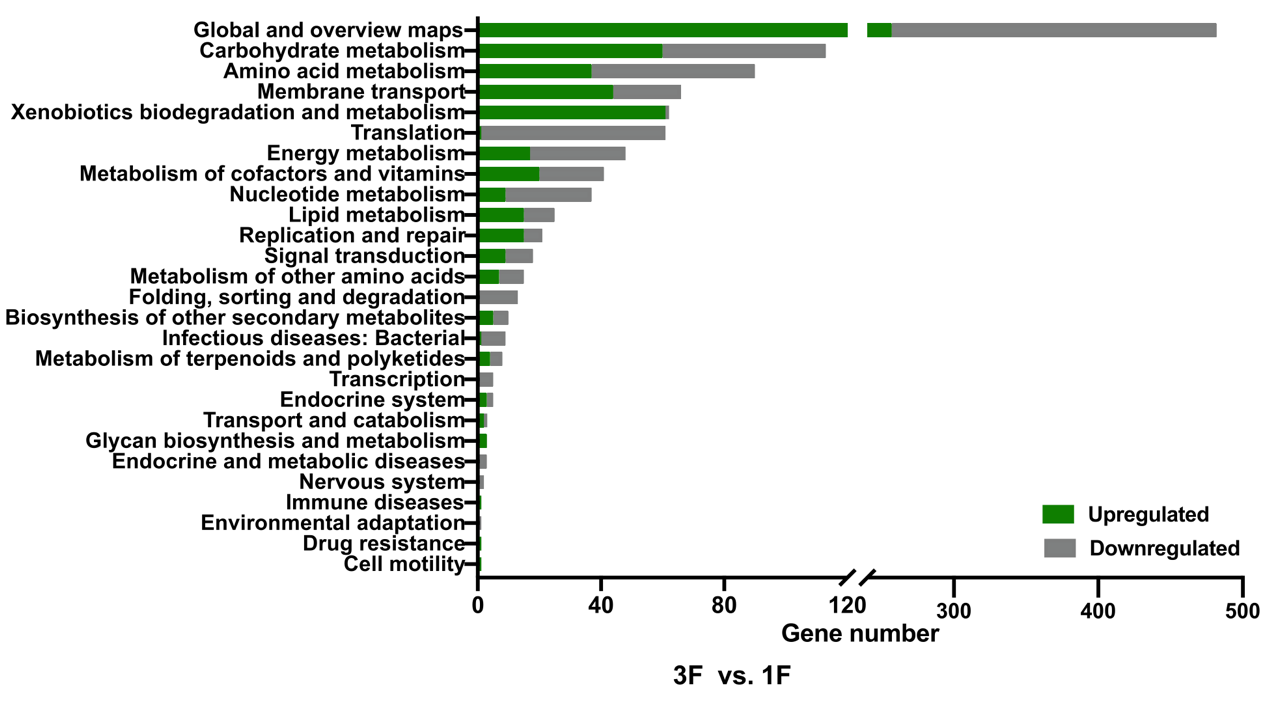
Supplementary Fig. 14.** Functional classification of transcriptome differences based on KEGG_small_class annotation in 3F vs. 1F. Upregulated and downregulated genes are indicated with green and gray, respectively. 1F, FM-1 cultivated with formaldehyde stress. 3F, FM-3 cultivated with formaldehyde stress. Three biological replicates were performed.


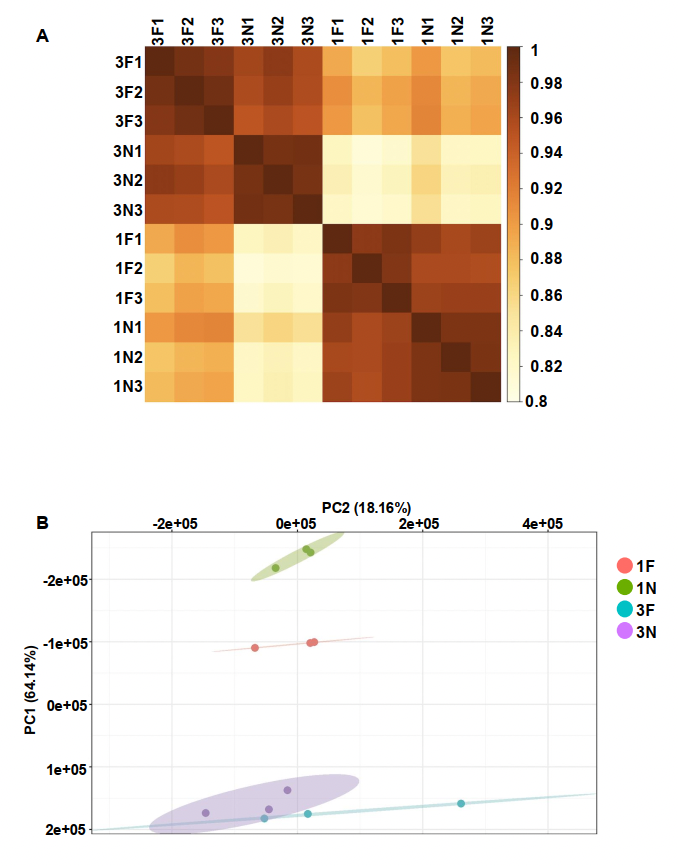


**Supplementary Fig. 15.** Evaluation of the accuracy and repeatability of proteome analysis. (A) Pearson’s correlation coefficient test. (B) Principal component analysis (PCA). Three biological replicates were performed. 1F, FM-1 cultivated with formaldehyde stress. 1N, FM-1 cultivated without formaldehyde stress. 3F, FM-3 cultivated with formaldehyde stress. 3N, FM-3 cultivated without formaldehyde stress. Three biological replicates were performed.

**
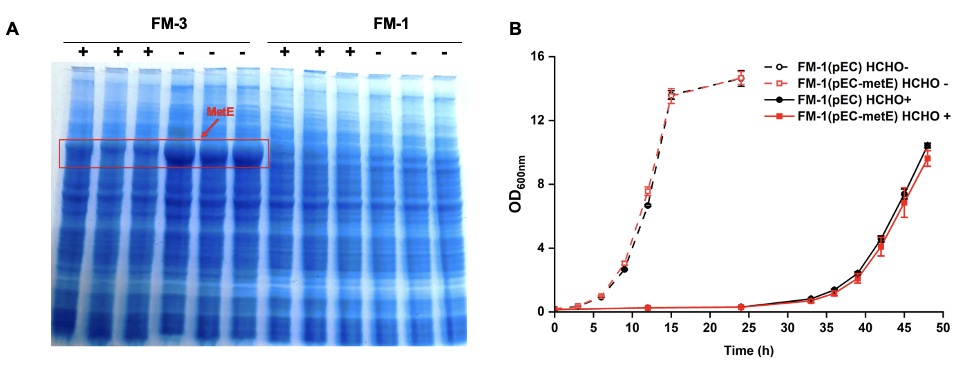
**

**Supplementary Fig. 16.** Effect of MetE on formaldehyde tolerance of FM-1. (A) SDS-PAGE of intracellular total proteins. (+): 0.8 mM formaldehyde, (-): 0 mM formaldehyde. (B) Effects of *metE* overexpression on formaldehyde tolerance. Strains were grown in CGXII minimal medium supplemented with 10 g/L glucose as carbon source with or without 1 mM formaldehyde.

**Supplementary Table 1.** Strains and plasmids used in this study

| **Strain or plasmid** | **Description^a^** | **Reference or source** |
| --- | --- | --- |
| **Strain** |  |  |
| *E. coli* |  |  |
| Trans1-T1 | General cloning host | TransGen Biotech |
| *C. glutamicum* ATCC 13032 | Wild-type strain | ATCC |
| FM-1 | Derivative of strain ATCC 13032 with its *ald* and *adhE* gene deleted | (1) |
| FM-2 | Mutant of strain FM-1 that tolerates high concentrations of formaldehyde | This study |
| FM-3 | Mutant of strain FM-1 that tolerates high concentrations of formaldehyde | This study |
| FM-4 | Mutant of strain FM-1 that tolerates high concentrations of formaldehyde | This study |
| FM-5 | Mutant of strain FM-1 that tolerates high concentrations of formaldehyde | This study |
| FM-1-*cgl0978*^686insG^ | Derivative of strain FM-1 harboring *cgl0978*^686insG^ mutation | This study |
| FM-1-*cgl2495*^G6163T^ | Derivative of strain FM-1 harboring *cgl2495*^G6163T^ mutation | This study |
| FM-1-*cgl0978*^G835T^ | Derivative of strain FM-1 harboring *cgl0978*^G835T^ mutation | This study |
| FM-1-*cgl2495*^G6160T^ | Derivative of strain FM-1 harboring *cgl2495*^G6160T^ mutation | This study |
| FM-1-*cgl0279*^323insG^ | Derivative of strain FM-1 harboring *cgl0279*^323insG^ mutation | This study |
| FM-1-*cgl0194*^T506A^ | Derivative of strain FM-1 harboring *cgl0194*^T506A^ mutation | This study |
| FM-1-*cgl11070*^T98C^ | Derivative of strain FM-1 harboring *cgl11070*^T98C^ mutation | This study |
| FM-1-*cgl0978*^G388T^ | Derivative of strain FM-1 harboring *cgl0978*^G388T^ mutation | This study |
| FM-1-*cgl11070*^T347A^ | Derivative of strain FM-1 harboring *cgl11070*^T347A^ mutation | This study |
| FM-1-*cgl0194*^G971A^ | Derivative of strain FM-1 harboring *cgl0194*^G971A^ mutation | This study |
| FM-1-*cgl2303*^G779T^ | Derivative of strain FM-1 harboring*cgl2303*^G779T^ mutation | This study |
| FM-1-*cgl2482*^G795T^ | Derivative of strain FM-1 harboring*cgl2482*^G795T^ mutation | This study |
| FM-1-*cgl2030*^C535T^ | Derivative of strain FM-1 harboring *cgl2030*^C535T^ mutation | This study |
| FM-1-*cgl3085*^C1277A^ | Derivative of strain FM-1 harboring *cgl3085*^C1277A^ mutation | This study |
| FM-1-*cgl2303*^G624T^ | Derivative of strain FM-1 harboring *cgl2303*^G624T^ mutation | This study |
| FM-1-*cgl1369*^G544T^ | Derivative of strain FM-1 harboring*cgl1369*^G544T^ mutation | This study |
| FM-1-*cgl2482*^A526T^ | Derivative of strain FM-1 harboring *cgl2482*^A526T^ mutation | This study |
| FM-1-*cgl1369*^A1198T^ | Derivative of strain FM-1 harboring *cgl1369*^A1198T^ mutation | This study |
| FM-1-*cgl1369*^C648A^ | Derivative of strain FM-1 harboring *cgl1369*^C648A^ mutation | This study |
| FM-1-*cgl1199*^T1226A^ | Derivative of strain FM-1 harboring *cgl1199*^T1226A^ mutation | This study |
| FM-1-*cgl1373*^2278insC^ | Derivative of strain FM-1 harboring *cgl1373*^2278 insC^ mutation | This study |
| FM-1-*cgl1590*^750insG^ | Derivative of strain FM-1 harboring *cgl1590*^750insG^ mutation | This study |
| FM-1-*cgl1590*^535insC^ | Derivative of strain FM-1 harboring *cgl1590*^535insC^ mutation | This study |
| FM-1-*cgl0752*^G761T^ | Derivative of strain FM-1 harboring *cgl0752*^G761T^ mutation | This study |
| FM-1-*cgl0942*^G443T^ | Derivative of strain FM-1 harboring *cgl0942*^G443T^ mutation | This study |
| FM-1-*cgl1199*^1015-1032del^ | Derivative of strain FM-1 harboring *cgl1199*^1015-1032del^ mutation | This study |
| FM-1-pEC-*cgl1590* | Derivative of strain FM-1 harboring pEC-XK99E-*cgl1590* | This study |
| FM-1-pEC-XK99E | Derivative of strain FM-1 harboring pEC-XK99E | This study |
| FM-1-Δ*cgl1590* | Derivative of strain FM-1 with *cgl1590* knocked out | This study |
| FM-1-*cgl1590*^754-1023del^ | Derivative of strain FM-1 with nucleotides 754-1032 were deleted | This study |
| FM-1-*cgl1590*^541-1023del^ | Derivative of strain FM-1 with nucleotides 541-1032 were deleted | This study |
| **Plasmid** |  |  |
| pEC-XK99E | Expression vector, IPTG-inducible promoter *P_trc_*, Km^R^ | (2) |
| pXMJ19 | Expression vector, IPTG-inducible promoter *P_tac_*, Cm^R^⁠ | (3) |
| pCas9 | pXMJ19 derivative carrying Cas9 gene, driven by IPTG-inducible promoter P*_tac_* | (4) |
| pnCas9(D10A)-AID-gRNA-*ccdB*^TS^ | pXMJ19 derivative carrying nCas9(D10A)-AID controlled by IPTG-inducible promoter P*_tac_* and gRNA-*ccdB* cassette, temperature sensitive | (5) |
| pRecT | pEC-XK99E derivative with *per* gene deleted, carrying a *recT* cassette controlled by constitutive promoter P*_ddh*_* | (5) |
| pK18*mobsacB* | Suicide vector for genome editing in *C. glutamicum*, *mob*, *sacB*, Km^R^ | (6) |
| pK18-*cgl0978*^686insG^ | pK18*mobsacB* derivative for introducing *cgl0978*^686insG^ mutation | This study |
| pK18-*cgl2495*^G6163T^ | pK18*mobsacB* derivative for introducing *cgl2495*^G6163T^ mutation | This study |
| pK18-*cgl0978*^G835T^ | pK18*mobsacB* derivative for introducing *cgl0978*^G835T^ mutation | This study |
| pK18-*cgl2495*^G6160T^ | pK18*mobsacB* derivative for introducing *cgl2495*^G6160T^ mutation | This study |
| pK18-*cgl0279*^323insG^ | pK18*mobsacB* derivative for introducing *gl0279*^323insG^ mutation | This study |
| pK18-*cgl0194*^T506A^ | pK18*mobsacB* derivative for introducing *cgl0194*^T506A^ mutation | This study |
| pK18-*cgl1070*^T98C^ | pK18*mobsacB* derivative for introducing *cgl11070*^T98C^ mutation | This study |
| pK18-*cgl0978*^G388T^ | pK18*mobsacB* derivative for introducing *cgl0978*^G388T^ mutation | This study |
| pK18-*cgl1070*^T347A^ | pK18*mobsacB* derivative for introducing *cgl11070*^T347A^ mutation | This study |
| pK18-*cgl0194*^G971A^ | pK18*mobsacB* derivative for introducing *cgl0194*^G971A^ mutation | This study |
| pK18-*cgl2303*^G779T^ | pK18*mobsacB* derivative for introducing *cgl2303*^G779T^ mutation | This study |
| pK18-*cgl2482*^G795T^ | pK18*mobsacB* derivative for introducing *cgl2482*^G795T^ mutation | This study |
| pK18-*cgl2030^C535^*^T^ | pK18*mobsacB* derivative for introducing *cgl2030^C535^*^T^ mutation | This study |
| pK18-*cgl2303*^G779T^ | pK18*mobsacB* derivative for introducing *cgl2303*^G779T^ mutation | This study |
| pK18-*cgl2482*^G795T^ | pK18*mobsacB* derivative for introducing *cgl2482*^G795T^ mutation | This study |
| pK18-*cgl2482*^G795T^ | pK18*mobsacB* derivative for introducing *cgl2482*^G795T^ mutation | This study |
| pK18-*cgl2030^C535^*^T^ | pK18*mobsacB* derivative for introducing *cgl2030^C535^*^T^ mutation | This study |
| pK18-*cgl3085*^C1277A^ | pK18*mobsacB* derivative for introducing *cgl3085*^C1277A^ mutation | This study |
| pK18-*cgl2303*^G624T^ | pK18*mobsacB* derivative for introducing *cgl2303*^G624T^ mutation | This study |
| pK18-*cgl1369*^G544T^ | pK18*mobsacB* derivative for introducing *cgl1369*^G544T^ mutation | This study |
| pK18-*cgl2482*^A526T^ | pK18*mobsacB* derivative for introducing *cgl2482*^A526T^ mutation | This study |
| pK18-*cgl1369*^A1198T^ | pK18*mobsacB* derivative for introducing *cgl1369*^A1198T^ mutation | This study |
| pK18-*cgl1369*^C648A^ | pK18*mobsacB* derivative for introducing *cgl1369*^C648A^ mutation | This study |
| pK18-*cgl1199*^T1226A^ | pK18*mobsacB* derivative for introducing *cgl1199*^T1226A^ mutation | This study |
| pK18-*cgl1373*^2278insC^ | pK18*mobsacB* derivative for introducing *cgl1373*^2278insC^ mutation | This study |
| pK18-*cgl1590*^750insG^ | pK18*mobsacB* derivative for introducing *cgl1590*^750insG^ mutation | This study |
| pK18-*cgl1590*^535insC^ | pK18*mobsacB* derivative for introducing *cgl1590*^535insC^ mutation | This study |
| pCas9gRNA-*cgl0942* | pnCas9（D10A）-AID-gRNA-*ccdB*^TS^ derivative for replacing *ccdB* with *cgl0942* gRNA | This study |
| pCas9gRNA-*cgl0752* | pnCas9（D10A）-AID-gRNA-*ccdB*^TS^ derivative for replacing *ccdB* with *cgl0752* gRNA | This study |
| pCas9gRNA*-cgl1199* | pnCas9（D10A）-AID-gRNA-*ccdB*TS derivative for replacing *ccdB* with *cgl1199* gRNA | This study |
| pK18-Δ*cgl1590* | pK18*mobsacB* derivative for knocking out *cgl1590* gene | This study |
| pK18-*cgl1590*^754-1023del^ | pK18*mobsacB* derivative for knocking out the 754-1023 bp of *cgl1590* gene | This study |
| pK18-*cgl1590*^541-1023del^ | pK18*mobsacB* derivative for knocking out the 541-1023 bp of *cgl1590* gene | This study |

^a^Km^R^ and Cm^R^ represent resistance to kanamycin, and chloramphenicol, respectively.

**Supplementary Table 2.** Primers used in this study

| **Primers** | **Sequence（5’-3’）** | **Relevance** |
| --- | --- | --- |
| *cgl0978*^686insG^-F1 | GAGCTCGGTACCCGGGGATCCCGACCCACCGACTGAAACC | pK18-*cgl0978*^686insG^ |
| *cgl0978*^686insG^-R1 | GATTCTTTGGGGGGCTCG |  |
| *cgl0978*^686insG^-F2 | CGAGCCCCCCAAAGAATC |  |
| *cgl0978*^686insG^-R2 | CAGGTCGACTCTAGAGGATCCGGATCGCAGATGTGCCTAAAA |  |
| *cgl2495*^G6163T^-F1 | GAGCTCGGTACCCGGGGATCCTCGTCCATGCAAGTTCGATG | pK18-*cgl2495*^G6163T^ |
| *cgl2495*^G6163T^-R1 | CGGCGTCAGTCGTCGAAA |  |
| *cgl2495*^G6163T^-F2 | TTTCGACGACTGACGCCG |  |
| *cgl2495*^G6163T^-R2 | CAGGTCGACTCTAGAGGATCCATTCCATCGAGACCCTGGTT |  |
| *cgl0978*^G835T^-F1 | GAGCTCGGTACCCGGGGATCCCGTTGACCTGCCTGAAGACC | pK18-*cgl0978*^G835T^ |
| *cgl0978*^G835T^-R1 | TGCCGCATCACTCGCCTC |  |
| *cgl0978*^G835T^-F2 | GAGGCGAGTGATGCGGCA |  |
| *cgl0978*^G835T^-R2 | CAGGTCGACTCTAGAGGATCCTGTCATCACCGCCCAACAAC |  |
| *cgl2495*^G6160T^-F1 | GAGCTCGGTACCCGGGGATCCTGGTCGGTAAGGAGTTGGCA | pK18-*cgl2495*^G6160T^ |
| *cgl2495*^G6160T^-R1 | AACTCGATCGCGTCGGCA |  |
| *cgl2495*^G6160T^-F2 | TGCCGACGCGATCGAGTT |  |
| *cgl2495*^G6160T^-R2 | CAGGTCGACTCTAGAGGATCCTCCATCGAGACCCTGGTTGAG |  |
| *cgl0279*^323insG^-F1 | GAGCTCGGTACCCGGGGATCCGGGAATCCACCAGGCAAGCT | pK18-*cgl0279*^323insG^ |
| *cgl0279*^323insG^-R1 | ATCTCGCCCCCCCTGAGC |  |
| *cgl0279*^323insG^-F2 | GCTCAGGGGGGGCGAGAT |  |
| *cgl0279*^323insG^-R2 | CAGGTCGACTCTAGAGGATCCATCATCGCCATCGCAATCAC |  |
| *cgl0194*^T506A^-F1 | GAGCTCGGTACCCGGGGATCCGCTGGTGACGTGGCTGTTGC | pK18-*cgl0194*^T506A^ |
| *cgl0194*^T506A^-R1 | GAGCCGCCGTTGCACAGC |  |
| *cgl0194*^T506A^-F2 | GCTGTGCAACGGCGGCTC |  |
| *cgl0194*^T506A^-R2 | CAGGTCGACTCTAGAGGATCCGGGTGAGCAGCACGAAGTAGAGTA |  |
| *cgl11070*^T98C^-F1 | GAGCTCGGTACCCGGGGATCCCGGGCAAGTTGGAAAGAGTCAC | pK18-*cgl11070*^T98C^ |
| *cgl11070*^T98C^-R1 | CCCCTTCGCTCCGCGCAC |  |
| *cgl11070*^T98C^-F2 | GTGCGCGGAGCGAAGGGG |  |
| *cgl11070*^T98C^-R2 | CAGGTCGACTCTAGAGGATCCTGATTCCACGCCACTTGGTTCG |  |
| *cgl0978*^G388T^-F1 | GAGCTCGGTACCCGGGGATCCAACCCAGTTGTTGATCCAGG | pK18-*cgl0978*^G388T^ |
| *cgl0978*^G388T^-R1 | AAGCATTTGAGTCAGCTCAAAC |  |
| *cgl0978*^G388T^-F2 | GTTTGAGCTGACTCAAATGCTT |  |
| *cgl0978*^G388T^-R2 | CAGGTCGACTCTAGAGGATCCTAACCTCGTCGCACAAACC |  |
| *cgl11070*^T347A^-F1 | GAGCTCGGTACCCGGGGATCCGGGGAAGAACAGGACTCGT | pK18-*cgl11070*^T347A^ |
| *cgl11070*^T347A^-R1 | GGAGAAAACGACCAGTGGTT |  |
| *cgl11070*^T347A^-F2 | AACCACTGGTCGTTTTCTCC |  |
| *cgl11070*^T347A^-R2 | CAGGTCGACTCTAGAGGATCCACGGTGCTTGAGGCGAAT |  |
| *cgl0194*^G971A^-F1 | GAGCTCGGTACCCGGGGATCCCGCTTGACACCGAATAGGA | pK18-*cgl0194*^G971A^ |
| *cgl0194*^G971A^-R1 | AATGTCCAGGTGATCCACG |  |
| *cgl0194*^G971A^-F2 | CGTGGATCACCTGGACATT |  |
| *cgl0194*^G971A^-R2 | CAGGTCGACTCTAGAGGATCCGATATGGGCGACGTTTGTG |  |
| *cgl2303*^G779T^-F1 | GAGCTCGGTACCCGGGGATCCGCAAGCACTGCGATGACC | pK18-*cgl2303*^G779T^ |
| *cgl2303*^G779T^-R1 | GGTTTTCGACGTTGGTGC |  |
| *cgl2303*^G779T^-F2 | GCACCAACGTCGAAAACC |  |
| *cgl2303*^G779T^-R2 | CAGGTCGACTCTAGAGGATCCGATCAAACCAGAAGAATCCGT |  |
| *cgl2482*^G795T^-F1 | GAGCTCGGTACCCGGGGATCCTTTCCTTCAGTGGCATTGTC | pK18-*cgl2482*^G795T^ |
| *cgl2482*^G795T^-R1 | AGACCGCGTTGAAAAAATC |  |
| *cgl2482*^G795T^-F2 | GATTTTTTCAACGCGGTCT |  |
| *cgl2482*^G795T^-R2 | CAGGTCGACTCTAGAGGATCCTCAGCCCAGGATTTATTAGGA |  |
| *cgl2030^C535^*^T^-F1 | GAGCTCGGTACCCGGGGATCCTGGTCAAGATTGGGCTGC | pK18-*cgl2030^C535^*^T^ |
| *cgl2030^C535^*^T^-R1 | GTTGCCTCAGTCTGGACTATTC |  |
| *cgl2030^C535^*^T^-F2 | GAATAGTCCAGACTGAGGCAAC |  |
| *cgl2030^C535^*^T^-R2 | CAGGTCGACTCTAGAGGATCCGTTTGAAGCCCGCGATAA |  |
| *cgl3085*^C1277A^-F1 | GAGCTCGGTACCCGGGGATCCGGCGTAGGACTCGTCGGTA | pK18-*cgl3085*^C1277A^ |
| *cgl3085*^C1277A^-R1 | GTGAAGTTGACATCGCAGAGC |  |
| *cgl3085*^C1277A^-F2 | GCTCTGCGATGTCAACTTCAC |  |
| *cgl3085*^C1277A^-R2 | CAGGTCGACTCTAGAGGATCC CCGATGGTGAATTAACTTTCC |  |
| *cgl2303*^G624T^-F1 | GAGCTCGGTACCCGGGGATCCCCCGCAAGATCGACAACC | pK18-*cgl2303*^G624T^ |
| *cgl2303*^G624T^-R1 | AGAAGCGGTGACAGAAGTACTG |  |
| *cgl2303*^G624T^-F2 | CAGTACTTCTGTCACCGCTTCT |  |
| *cgl2303*^G624T^-R2 | CAGGTCGACTCTAGAGGATCC TGCCTGAGGATATTTGCTCAT |  |
| *cgl1369*^G544T^-F1 | GAGCTCGGTACCCGGGGATCC CATCCAGCTAAATCCTAACTATGG | pK18-*cgl1369*^G544T^ |
| *cgl1369*^G544T^-R1 | TCAACACAAAGGAACGGTCA |  |
| *cgl1369*^G544T^-F2 | TGACCGTTCCTTTGTGTTGA |  |
| *cgl1369*^G544T^-R2 | CAGGTCGACTCTAGAGGATCCGTATCAATATCTGAGTGCAGGTAGC |  |
| *cgl2482*^A526T^-F1 | GAGCTCGGTACCCGGGGATCCTACTCGGGTGAGATCAAGAACA | pK18-*cgl2482*^A526T^ |
| *cgl2482*^A526T^-R1 | GCCCTGTGCTCCGTTAAC |  |
| *cgl2482*^A526T^-F2 | GTTAACGGAGCACAGGGC |  |
| *cgl2482*^A526T^-R2 | CAGGTCGACTCTAGAGGATCCCAGAAAGCCTCGCCTGTT |  |
| *cgl1369*^A1198T^-F1 | GAGCTCGGTACCCGGGGATCCGGGTGCTACTGGTACGGG | pK18-*cgl1369*^A1198T^ |
| *cgl1369*^A1198T^-R1 | TCCTCCCAGGACAATGGG |  |
| *cgl1369*^A1198T^-F2 | CCCATTGTCCTGGGAGGA |  |
| *cgl1369*^A1198T^-R2 | CAGGTCGACTCTAGAGGATCCCGCTCATGTGACCTTCCC |  |
| *cgl1369*^C648A^-F1 | GAGCTCGGTACCCGGGGATCCGCCGACTTGATTGAGGAGG | pK18-*cgl1369*^C648A^ |
| *cgl1369*^C648A^-R1 | TTCACGCGTAAAGCACCA |  |
| *cgl1369*^C648A^-F2 | TGGTGCTTTACGCGTGAA |  |
| *cgl1369*^C648A^-R2 | CAGGTCGACTCTAGAGGATCCCCAATGGTCTGAATCAGTGAG |  |
| *cgl1199*^T1226A^-F1 | GAGCTCGGTACCCGGGGATCCGGTTGAGCAAGCACAAGAAGC | pK18-*cgl1199*^T1226A^ |
| *cgl1199*^T1226A^-R1 | ATACGGCGGTTCAGCTGG |  |
| *cgl1199*^T1226A^-F2 | CCAGCTGAACCGCCGTAT |  |
| *cgl1199*^T1226A^-R2 | CAGGTCGACTCTAGAGGATCCATGGCTTGCTGATTATCAAGTG |  |
| *cgl1373*^2278insC^-F1 | GAGCTCGGTACCCGGGGATCCGATAGTCTTCTAAACAAGGGCGTC | pK18-*cgl1373*^2278insC^ |
| *cgl1373*^2278insC^-R1 | AGAATCCCCCCCAGGGAT |  |
| *cgl1373*^2278insC^-F2 | ATCCCTGGGGGGGATTCT |  |
| *cgl1373*^2278insC^-R2 | CAGGTCGACTCTAGAGGATCCTGCTTTGCTGCGTGGTGA |  |
| *cgl1590*^750insG^-F1 | GAGCTCGGTACCCGGGGATCCGTTCGACTTTCCCGCTTGAT | pK18-*cgl1590*^750insG^ |
| *cgl1590*^750insG^-R1 | TCCGAGCCAGGGGGAGAC |  |
| *cgl1590*^750insG^-F2 | GTCTCCCCCTGGCTCGGA |  |
| *cgl1590*^750insG^-R2 | CAGGTCGACTCTAGAGGATCCGAGTGGACAAGCCAGTATCTGACTA |  |
| *cgl1590*^535insC^-F1 | GAGCTCGGTACCCGGGGATCCTCCGCTGACCACAGAAGGC | pK18-*cgl1590*^535insC^ |
| *cgl1590*^535insC^-R1 | TAGCTGCAACCCCCCGGG |  |
| *cgl1590*^535insC^-F2 | CCCGGGGGGTTGCAGCTA |  |
| *cgl1590*^535insC^-R2 | CAGGTCGACTCTAGAGGATCCAACCGTGCTGTCTCCTGTGAA |  |
| *cgl0752*-gF | TTCAGCCACTGACATGCTCATGGG | pCas9gRNA-*cgl0752* |
| *cgl0752*-gR | AAACCCCATGAGCATGTCAGTGGC |  |
| *cgl0942*-gF | TTCACAGATCCAGGGCTTCTTCGA | pCas9gRNA-*cgl0942* |
| *cgl0942*-gR | AAACTCGAAGAAGCCCTGGATCTG |  |
| *cgl1199*-gF | TTCAGGCGATTGCGGCGTCCGCGA | pCas9gRNA*-cgl1199* |
| *cgl1199*-gR | AAACTCGCGGACGCCGCAATCGCC |  |
| *cgl0752*^G761T^ | CCTGATCGACGGCATCAACCGCGCCACTGACATGCTCATGGGCGTCAAGAACGTGCTTGTCTGCGGTTACGGCGATGTCGGCAAGGGCT | Mutation at 761 (G to T) of *cgl0752* |
| *cgl0942*^G443T^ | TGTGATCGGTGAGGATCGGCATGGCGTGCATGTGATCGACTGGGACGTCGAAGAAGCCCTGGATCTGATCGGTGTGCAAGTCCACGGAC | Mutation at 443 (G to T) of *cgl0942* |
| *cgl1199*^1015-1032del^ | AATGACCGCGACAACGACGACAACGATGATCGCCGCAACCGTCGCGGACGCAACGACCGTAACGATCGCGACAACCGAGATAACCGGGATAACCG | Mutation at 1015 (1015-1032del) of *cgl1199* |
| Δ*cgl1199*-F1 | GAGCTCGGTACCCGGGGATCCGTTATAGAGCATCGACGCCAC | pK18-Δ*cgl1199* |
| Δ*cgl1199*-R1 | GATCAGATCGCCCTTACGC |  |
| Δ*cgl1199*-F2 | GCGTAAGGGCGATCTGATCCGTCGTATTCTGTCTGCACTT |  |
| Δ*cgl1199*-R2 | CAGGTCGACTCTAGAGGATCCCTCTTCCTTCATCGCCTGTA |  |
| Δ*cgl1590*-F1 | GAGCTCGGTACCCGGGGATCCAGCCAAATGCTCAGGAACA | pK18-Δ*cgl1590* |
| Δ*cgl1590*-R1 | GCCAAGACACTGTCCTCACA |  |
| Δ*cgl1590*-F2 | TGTGAGGACAGTGTCTTGGCGCCACCATCATCAGCAACT |  |
| Δ*cgl1590*-R2 | CAGGTCGACTCTAGAGGATCCCTGATAAGTCTGCACAAGAAAATC |  |
| *cgl1590*^754-1023del^-F1 | GAGCTCGGTACCCGGGGATCCGTTCGCACTACGATCTGCTC | pK18-*cgl1590*^754-1023del^ |
| *cgl1590*^754-1023del^-R1 | TAAGGAGTAGGCGTGTCACTG |  |
| *cgl1590*^754-1023del^-F2 | CAGTGACACGCCTACTCCTTACCCTGGCTCGGACGTCAG |  |
| *cgl1590*^754-1023del^-R2 | CAGGTCGACTCTAGAGGATCCGAGTGGACAAGCCAGTATCTGACTA |  |
| *cgl1590*^541-1023del^-F1 | GAGCTCGGTACCCGGGGATCCGTTCGCACTACGATCTGCTC | pK18-*cgl1590*^541-1023del^ |
| *cgl1590*^541-1023del^-R1 | TAAGGAGTAGGCGTGTCACTG |  |
| *cgl1590*^541-1023del^-F2 | CAGTGACACGCCTACTCCTTAGGGGGTTGCAGCTACCGC |  |
| *cgl1590*^541-1023del^-R2 | CAGTCGACTCTAGAGGATCCGAGTGGACAAGCCAGTATCTGACTA |  |

**Supplementary Table 5.** Comparative transcriptomic and proteomic analysis of shared mutated genes in evolved strains.

| Gene ID | Gene name | Transcript level (log_2_(Fold change)) | | | | Protein abundance (log_2_(Fold change)) | | | |
| --- | --- | --- | --- | --- | --- | --- | --- | --- | --- |
|  |  | 1F vs 1N | 3F vs 3N | 3N vs 1N | 3F vs 1F | 1F vs 1N | 3F vs 3N | 3N vs 1N | 3F vs 1F |
| *cgl0978* | *tdcB* | / | / | 4.82 | 4.33 | / | / | / | / |
| *cgl2495* | - | / | / | -1.51 | -1.15 | / | / | / | / |
| *cgl0194* | - | / | 1.07 | / | / | / | / | / | / |
| *cgl1070* | - | / | / | -1.08 | / | / | / | / | / |
| *cgl2303* | - | / | / | -1.10 | / | / | / | / | / |
| *cgl1590* | - | / | / | -1.07 | / | / | / | / | / |
| *cgl0279* | ***whiB*** | / | / | / | / | / | / | / | / |
| *cgl1199* | *rho* | / | / | -1.33 | -2.14 | / | / | / | / |
| *cgl2030* | - | / | / | 5.60 | 4.29 | / | / | / | / |
| *cgl3085* | *pcnA* | / | / | 4.29 | / | / | / | / | / |
| *cgl1369* | *uvrB* | / | / | / | / | / | / | / | / |
| *cgl2482* | *glsK* | / | / | / | / | / | / | -1.14 | / |
| *cgl1373* | - | / | / | 1.60 | / | / | / | / | / |
| *cgl0752* | *sahH* | / | / | -1.33 | -1.11 | / | / | -1.42 | / |
| *cgl0942* | *prsA* | / | / | -1.52 | -1.90 | / | / | / | -1.32 |
| *cgl0754* | *mtrA* | / | / | -.22 | -1.50 | / | / | / | / |

**Supplementary Table 3.** Summary of mutations identified by whole-genome sequencing of FM-2, FM-3, FM-4 and FM-5 for Venn Diagram Analysis. Table S5 is provided in a separate Excel file.

**Supplementary Table 4.** Gene transcript level changes between the starting strain FM-1 and the evolved mutant FM-3 cultivated in CGXII minimal medium supplemented with or without 0.8 mM formaldehyde, and 10 g/L glucose was supplemented as the carbon source. 1F, FM-1 cultivated with formaldehyde stress. 1N, FM-1 cultivated without formaldehyde stress. 3F, FM-3 cultivated with formaldehyde stress. 3N, FM-3 cultivated without formaldehyde stress. Table S3 is provided in a separate Excel file.

**Supplementary Table 6.** Protein abundance differences between the starting strain FM-1 and the evolved mutant FM-3 cultivated in CGXII minimal medium supplemented with or without 0.8 mM formaldehyde, and 10 g/L glucose was supplemented as the carbon source. 1F, FM-1 cultivated with formaldehyde stress. 1N, FM-1 cultivated without formaldehyde stress. 3F, FM-3 cultivated with formaldehyde stress. 3N, FM-3 cultivated without formaldehyde stress. Table S4 is provided in a separate Excel file.

**References**

1. Tuyishime P, Wang Y, Fan L, Zhang Q, Li Q, Zheng P, Sun J, Ma Y. Engineering *Corynebacterium glutamicum* for methanol-dependent growth and glutamate production. Metab Eng 2018;49(220-231. <https://doi.org/10.1016/j.ymben.2018.07.011>.

2. Kirchner OT, A. . Tools for genetic engineering in the amino acid-producing bacterium *Corynebacterium glutamicum*. J Biotechnol 2003;104(287-299. <https://doi.org/10.1016/S0168-1656(03)00148-2>.

3. Jakoby MN-N, CE. Burkovski, A. Construction and application of new *Corynebacterium glutamicum* vectors. Biotechnol Tech 1999;13:437-441. <https://doi.org/10.1023/A:1008968419217>.

4. Liu J, Wang Y, Zheng P, Sun J. CRISPR/Cas9-mediated ssDNA recombineering in *Corynebacterium glutamicum*. Bio Protoc 2018;8(19):e3038. <https://doi.org/10.21769/BioProtoc.3038>.

5. Liu J, Liu M, Shi T, Sun G, Gao N, Zhao X, Guo X, Ni X, Yuan Q, Feng J, Liu Z, Guo Y, Chen J, Wang Y, Zheng P, Sun J. CRISPR-assisted rational flux-tuning and arrayed CRISPRi screening of an L-proline exporter for L-proline hyperproduction. Nat Commun 2022;13(1):891. <https://doi.org/10.1038/s41467-022-28501-7>.

6. Schäfer A, Tauch A, Jäger W, Kalinowski J, Thierbachb G, Pühler A. Small mobilizable multi-purpose cloning vectors derived from the *Escherichia coli* plasmids pK18 and pK19: selection of defined deletions in the chromosome of *Corynebacterium glutumicum* Gene 1994;145:69-73. <https://doi.org/10.1016/0378-1119(94)90324-7>.
